# Catalytic Amyloids: Turning Fibrils Into Biocatalysts

**DOI:** 10.1002/chem.202503095

**Published:** 2026-01-28

**Authors:** Alessandra Esposito, Linda Leone, Flavia Nastri, Angela Lombardi

**Affiliations:** ^1^ Department of Chemical Sciences University of Napoli Federico II Napoli Italy

**Keywords:** amyloids, biocatalysis, metalloenzymes, nanomaterials, peptides

## Abstract

Amyloids have been regarded as the pathological entities behind neurodegenerative diseases for a long time. The discovery that they also play physiological roles together with their ability to form stable and ordered scaffolds opened the door for their applications in different fields. In this context, catalytic amyloids have emerged as a new class of nanomaterials, merging the efficiency of enzymes with the robustness of heterogeneous catalysts. Indeed, these systems exploit the self‐assembly properties of amyloids while mimicking enzymatic functions by exposing catalytic moieties on their surface. In this review, we first provide an overview of the structural and functional properties of natural amyloids and their application in nanotechnology. Then, we survey the current state of art in the development of catalytic amyloids, based on bioinspired or de novo designed sequences. In both cases, the incorporation of specific functional groups provides the fibrils with catalytic functions. Finally, we illustrate the use of amyloid fibrils as platforms for enzyme immobilization. All the selected examples highlight the power of bridging amyloid structures and catalytic activities to shape innovative nanomaterials toward demanding needs.

## Graphical abstract

Amyloids, traditionally associated with diseases, have emerged as versatile catalytic scaffolds. From natural amyloid sequences to bioinspired and *de novo* designs, we highlight strategies to construct catalytic active sites and anchor enzymes onto fibrils, creating versatile nanomaterials with tunable activities.

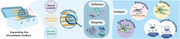

## 1 Introduction

Drawing inspiration from nature, protein nanotechnology leverages the unique ability of peptides and proteins to self‐assemble into a variety of nanoscale structures, performing specific functions [1]. For example, proteins such as collagen and silk fibroin naturally form fibrils that impart remarkable mechanical strength and structural support to animal body tissues and to cocoons, respectively [2, 3, 4, 5, 6]. Insights into these systems have driven the research toward the development of protein‐based nanomaterials with applications in several fields as biomaterials, medicine, biosensing [7, 8]. Among the diversity of nanostructures investigated, the hierarchical self‐assembly of amyloid fibrils gained increasing interest [9, 10]. Amyloid fibrils are highly ordered self‐assembled protein structures composed of long filaments that form insoluble aggregates, which are highly resistant to degradation [11]. This remarkable stability contributes to the formation of pathological aggregates linked to degenerative human diseases, commonly known as protein misfolding diseases (or protein conformational diseases) [12]. In turn, the misfolding and aggregation of proteins into amyloid fibrils is often linked to a number of currently incurable diseases, such as Alzheimer and Parkinson diseases, Type II diabetes, and spongiform encephalopathies [13]. Despite their widespread occurrence in neurodegenerative and non‐neuropathic diseases [12], the discovery that amyloids can also play functional roles in a variety of organisms has strongly stimulated researche to unravel the basis of their dual nature [14, 15, 16]. In parallel, attention has been devoted to the application of amyloid fibrils as templates or building blocks for the construction of ordered nanomaterials useful for biomedical [17], nanosensing [18, 19], and catalytic [20, 21, 22, 23, 24] applications. The structural features of amyloid fibrils, conferring exceptional mechanical strength and outstanding chemical stability, provide significant advantages [25], enabling the development of materials that exhibit unexpected biocompatibility and biodegradability. These characteristics have made them useful for many applications, such as drug delivery [26, 27, 28], functional hydrogels [29, 30, 31], and membranes [32, 33]. Besides their uses as novel polymers with specific structures, their repetitive architecture allows the introduction of additional functionality, such as, for example, catalytic sites that can be arranged in a regular pattern. Several studies have shown that native amyloid fibrils are able to promote catalytic reactions, stimulating research into the development of artificial catalytic amyloids. They emerged as a promising class of functional nanomaterials, which combine the advantages of enzymatic and heterogeneous catalysis into peptide‐based nanostructures [20, 21, 22, 23, 24, 34, 35].

The catalytic behaviors of amyloid fibrils, made up by natural proteins or synthetic peptide assemblies, have been recently highlighted in several reviews [1, 20, 21, 22, 23, 24, 25, 34, 36]. They represent a huge contribution, giving a comprehensive view of the assemblies studied to date in light of the catalyzed reactions [20]. Some also discuss the possible involvements of amyloid catalysis in prebiotic molecular evolution and in the onset of neurodegenerative disease [37, 38].

**FIGURE 1:**
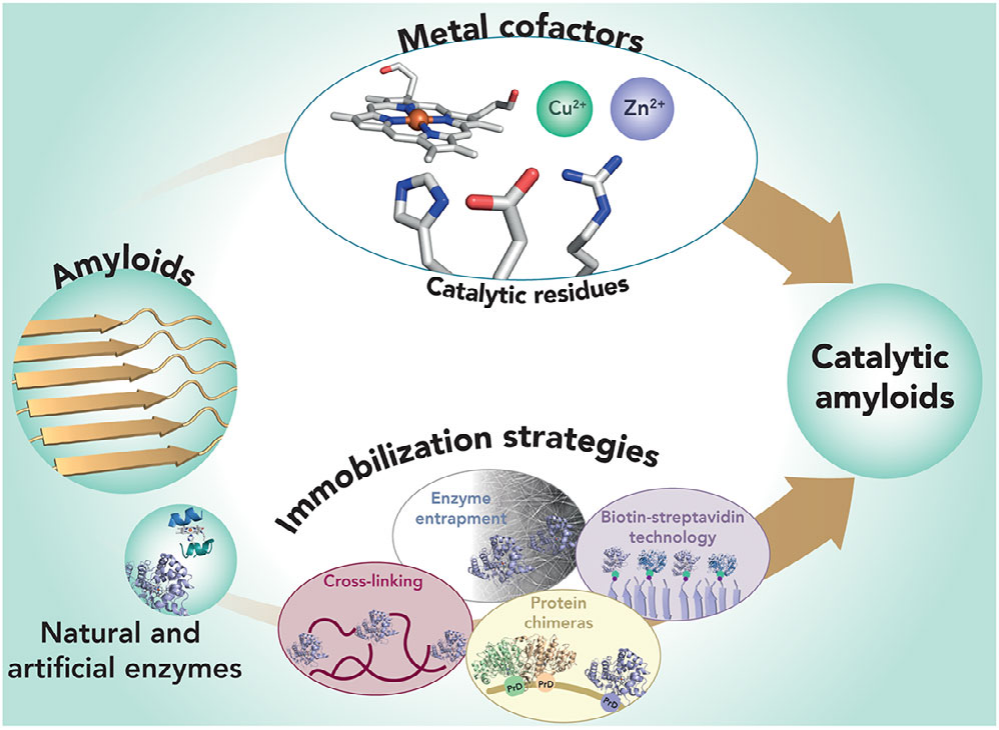
The goal of constructing catalytic amyloids can be achieved by several strategies and approaches. Starting from the selection of either natural, bioinspired, or de novo designed sequences, catalytic residues, cofactors, or enzymes can be incorporated into the amyloid fibrils. Finally, various immobilization strategies, including enzyme entrapment, biotin–streptavidin technology, cross‐linking, and protein chimeras, can be applied.

In this review, we intend to give our contribution to the field, mainly focusing on the different approaches and design strategies to obtain catalytically active amyloid fibril assemblies, involving either native or artificial peptide sequences (Figure 1). These approaches range from the incorporation of catalytic active sites directly into the amyloid fibers, either by using specific residues or metal cofactors [39], to the conjugation of whole biocatalysts to the amyloid structures. This can be accomplished either through covalent bonds, such as the construction of chimeric assemblies, or through non‐covalent interactions, as entrapment of enzymes into the fibrillar network or with biotin‐streptavidin technology (Figure 1) [20, 21]. All the above‐mentioned approaches have been effectively applied to two main categories of amyloid sequences, namely ‘bioinspired’ and ‘*de novo* designed’ [21]. Bioinspired catalytic amyloids refer to natural amyloid sequences in which specific modifications, as amino acid substitutions (*e.g*., unnatural amino acids or aromatic groups capable of π‐stacking) or incorporation of functional groups (*e.g*., alkyl tails), allow catalytic properties to be finely tuned and optimized toward defined reactions. Conversely, the *de novo* design approach foresees the design from the ground up of entirely new amyloid sequences, endowed with specific catalytic function.

To offer a helpful source of topics for the non‐specialist readership approaching the field of catalytic amyloids, in this review, we first describe the structural and mechanical behaviors of amyloid fibrils, highlighting the key properties that make them attractive for nanomaterial construction. In this respect, we provide an overview of how the inherent functional properties of natural amyloids have been exploited in biotechnological applications. Then, we illustrate the efforts made in the development of catalytic amyloids reported to date. Starting with naturally occurring examples, we move to artificial catalytic amyloids based on bioinspired and *de novo* designed sequences. Finally, we describe examples of catalytic amyloids developed by immobilizing natural and artificial enzymes on amyloid fibrils. We are aware that it is impossible to exhaustively cover the huge amount of work in the field. However, through selected examples, we intend to highlight how amyloid scaffolds represent a new tool in catalysis, offering customizable platforms for the fabrication of catalytic nanomaterials.

## 2 Structural and Mechanical Properties of Amyloid Fibrils

Amyloid fibrils are organized in a hierarchical structure in which structural subunits, defined as monomers, stacks symmetrically to afford structures on diverse length scales [40].

**FIGURE 2:**
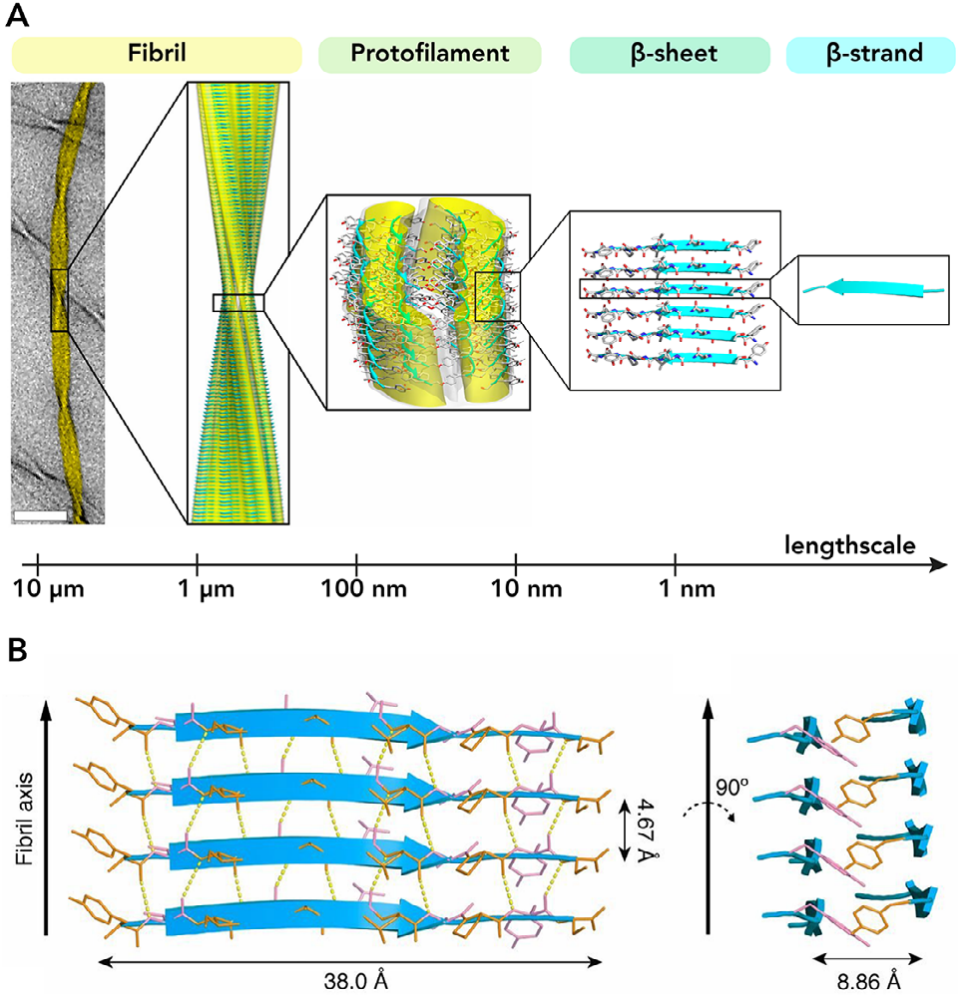
Hierarchical organization of amyloid fibrils [9]. (A) Amyloid fibrils (left TEM image (scale bar = 50 nm) and right cryo‐EM reconstruction) are formed by lateral association of protofilaments. In turn, protofilaments are formed by stacking of β‐sheets subunits that shows β‐strands aligned perpendicular to the fibril axis. (B) Perpendicular view of the β‐sheet to the fibril axis, highlighting the hydrogen bonds that stabilize the β‐sheet (represented by yellow lines). A cross‐sectional view of the two‐sheet protofilament along the direction of the peptide chain. Adapted with permission from Fitzpatrick, A. et al., PNAS *2013, 110*. Permission granted from PNAS.

**FIGURE 3:**
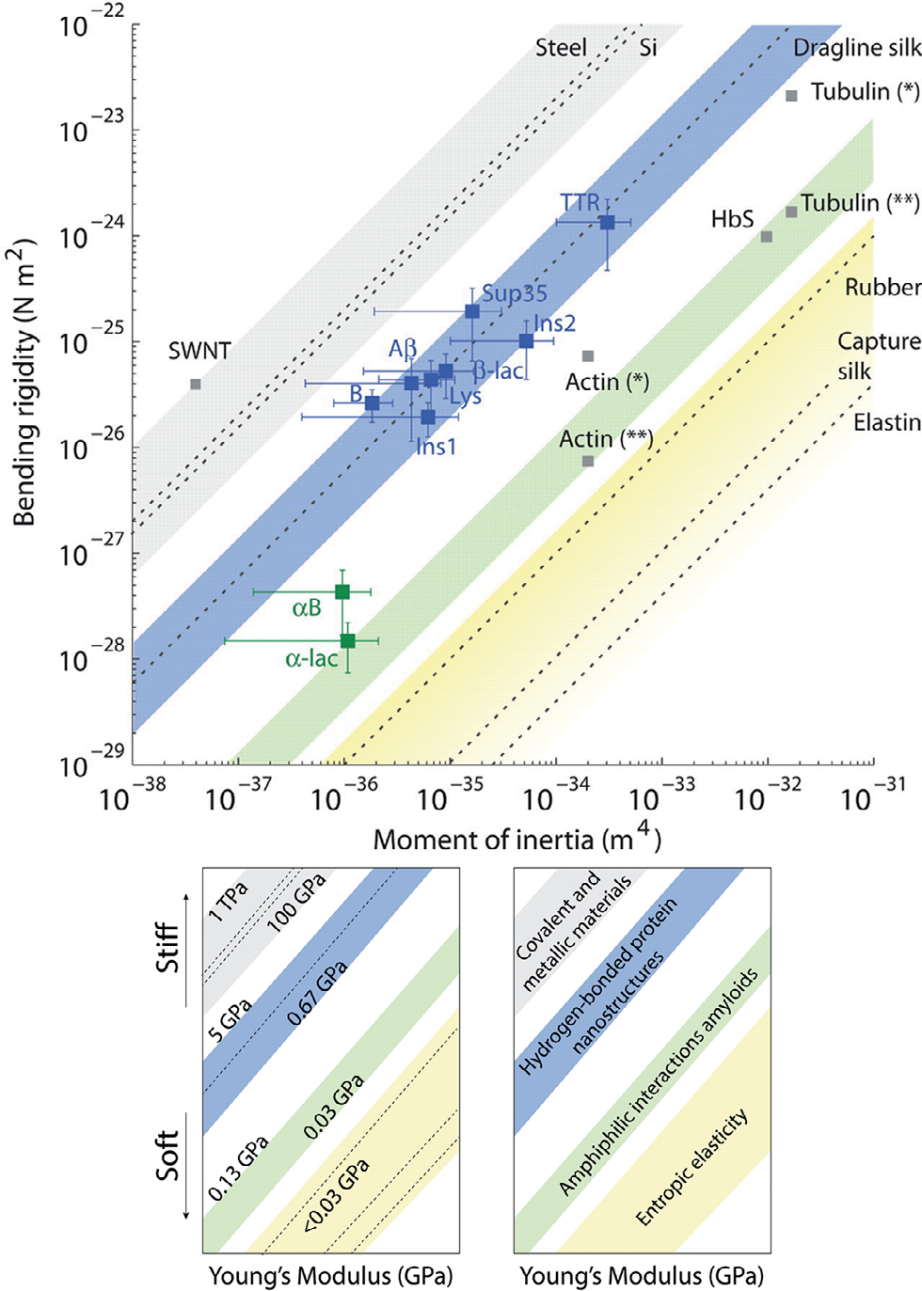
Comparison of different material classes based on elastic properties [5]. The blue squares show the bending rigidities of various amyloid fibrils, organized by their cross‐sectional moments of inertia, from TTR(105–115) and other amyloids like Sup35, β‐lactoglobulin (β‐lac), lysozyme (Lys), Aβ(1–42) peptide (Aβ), insulin single filament and 2 (Ins1 and Ins2, respectively), and insulin B chain (B) fibrils. The green squares represent less‐ordered protofibrils, such as α‐lactalbumin (α‐lac) and αB‐crystallin (αB). For comparison, data is also presented for single‐walled carbon nanotubes (SWNT), steel, silicon, silk, tubulin, sickle‐cell hemoglobin (HbS), actin, rubber, and elastin. Reproduced from Knowles, T.P., Fitzpatrick, A.W., Meehan, S., Mott, H.R., Vendruscolo, M., Dobson, C.M., and Welland, M.E., Science, 318, 1900–1903 (2007). AAAS.

The fibril is formed of aligned protofilaments composed of an array of several monomeric β‐strands arranged in a β‐sheet conformation (Figure 2A) [41, 42, 43]. Structural analysis revealed the common core architecture of amyloid, based on the formation of an inter‐backbone hydrogen‐bonding network between adjacent amino acids (Figure 2B) [44, 45]. In addition, van der Waals and hydrophobic interactions contribute significantly to lateral packing of protofilaments, to folding of their constituent subunits, and to the overall stability of the structure [40, 46]. Moreover, the β‐strands elongation along the fibrillar axis can be in either a parallel or antiparallel arrangement, depending on the orientation and alignment of the monomers. Amyloid fibril formation process involves a nucleation‐dependent polymerization mechanism, allowing for a rapid assembly once seeded by pre‐existing fibrils, making their growth highly controllable [47, 48, 49]. The 3D arrangement of the amyloid structures was first elucidated by X‐ray diffraction (XRD) [41, 50]. Advances in solid‐state NMR spectroscopy and the advent of cryo‐electron microscopy (cryo‐EM) strongly empowered the field by allowing analysis on previously undetectable samples [51]. Indeed, cryo‐EM revealed a remarkable diversity of amyloid structures, including tau filaments, amyloid‐β, and polymorphic strains in α‐synuclein and other amyloids [52]. Furthermore, high‐resolution cryo‐EM maps allow atomic modeling of β‐strand arrangements, inter‐sheet interactions, and post‐translational modifications, providing insight into aggregation mechanisms, toxicity, and potential therapeutic treatments. When packed closely together, the protofilaments follow a specific symmetry that results in mature fibrils displaying a wide variety of morphologies, from twisted ribbon‐like forms to helical rope‐like or flat tape‐like configurations [53, 54]. This feature highlights the polymorphic nature of amyloids, where differences in the number and arrangement of protofilaments give rise to various fibrillar architectures. More in detail, α‐synuclein fibrils exhibit helical ribbon structures both left‐ and right‐handed, depending on pH and other experimental conditions [55]. Likewise, Adamcik and coworkers analyzed the aggregation of heat‐denatured β‐lactoglobulin amyloid fibrils by AFM and theoretical models [56]. They discovered that mature fibrils have a left‐handed multistranded twisted shape with filament heights that increase gradually and persistence lengths that range from 1 to 4 µm. Additionally, with the increase of the number of filaments, the helical pitch of these nanofibrils increased. One of the most representative examples of the polymorphism and hierarchical assembly is reported by Fitzpatrick and coworkers [9], who extensively examined the atomic structure of fibrils formed by the TTR(105–115) peptide derived from human transthyretin. Under specific conditions, cryo‐EM analysis showed that protofilaments stacked in an antiparallel arrangement formed either singlet fibrils or intertwined filaments (*i.e*., from two to four), in turn forming a twisted ribbon mature fibril with periodical crossover distances. In recent years, innovative AI methods, such as AlphaFold2 [57], were developed to predict amyloidogenic sequences and models of fibril packing in order to complement the experimental cryo‐EM data. In line with that, also hybrid approaches that combine cryo‐EM, solid‐state NMR, and computational modeling, were used, enabling the study of amyloids in near‐native conditions, capturing polymorphic heterogeneity and dynamic structural transitions. One of the key features that make amyloid fibrils particularly appealing for their use as nanomaterials and scaffolds is their mechanical strength, often comparable to hard materials, as silk and steel (Figure 3) [3, 4, 5, 6, 58, 59]. Indeed, as reported in detail by Smith et al. [44], fibrils derived from insulin have a mechanical strength (evaluated by the Young's modulus value of 0.6 ± 0.4 GPa) comparable to that of steel (Young's modulus ranging from 0.6 to 1.8 GPa) and stiffness similar to silk (1–10 GPa). Apart from insulin, Knowles and coworkers [5] reported mechanical stiffness of amyloid fibrils formed by α‐lactalbumin, β‐lactoglobulin, and a fragment of the human transthyretin named TTR(105‐115). Intriguingly, the bending rigidities of these amyloid fibrils differ significantly, spanning nearly four orders of magnitude, from highly flexible α‐lactalbumin to extremely stiff TTR(105‐115). The explanation of this behavior can be ascribed to different stabilizing inter‐protofilament interaction between peptide backbones. This remarkably high breaking strength is particularly surprising given that amyloid lack of covalent interaction in the structure. Indeed, fibril stability and Young's modulus are enhanced by non‐covalent interactions between side chains, such as hydrophobic forces and additional hydrogen bonds. Self‐assembled peptide fibrils exhibit remarkable stability across a wide range of environmental conditions. They remain stable under varying pH values and salt concentrations [60], higher pressure (up to 1.3 GPa) [61], and to thermic and proteolytic degradation. All the features described above makes amyloids fibrils, either derived from natural or engineered proteins, attractive as nanomaterials for a plethora of technological and biological applications, spanning from biosensing to catalysis, as well as electronics or cellular scaffolds [36, 62, 63].

## 3 Beyond Diseases: Functional Amyloids and Their Application in Nanotechnology

The discovery that amyloids also play physiological and beneficial roles in different organisms changed the research perspectives on these protein structures [16, 64, 65, 66]. They were found to be involved in the bacteria biofilm formation [67, 68, 69, 70], in the production of melanin [71], and in peptide hormone storage and release [72], as well as they act as protective materials in plant seeds [73] and insect eggshells [74]. These discoveries have been a source of inspiration for the design of novel nanostructured materials [75, 76, 77]. Indeed, amyloid fibrils, due to their ability to form complex higher‐order structures as gels, film, and membranes [78, 79, 80, 81, 82, 83, 84, 85, 86], useful for trapping small molecules [87, 88, 89] or drugs [90, 91], have proven to be particularly versatile for a plethora of biotechnological applications.

**FIGURE 4:**
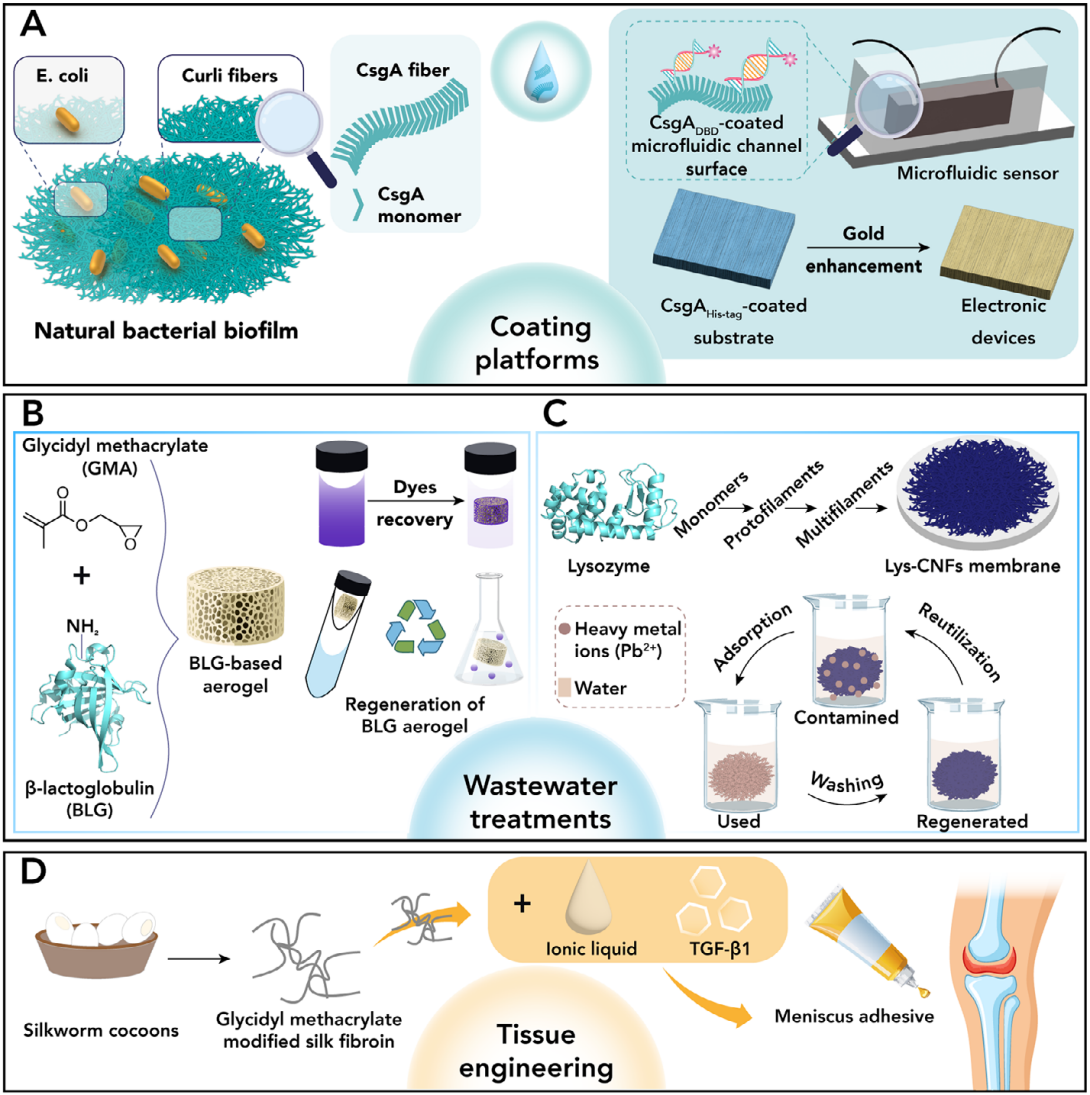
Modified amyloid fibrils for the construction of functional nanomaterials. (A) *E. coli* biofilms engineered to produce self‐assembled CsgA nanofibers, which can be modularly functionalized to create protein coatings for applications such as electronic devices and microfluidic sensors [78]. Protein‐based reusable materials for wastewater treatment (B–C), in particular (B) β‐lactoglobulin aerogels for organic pollutant adsorption and reuse [79] and (C) lysozyme membranes for rapid Pb(II) removal and regeneration [82]. (D) Composite hydrogel adhesive for meniscus repair, based on modified silk fibroin (SFMA) extracted from silkworm cocoons, phenylboronic acid–ionic liquid (PIL), and a growth factor (TGF‐β1) [85]. (Panel (A) Reproduced from Li et al., Sci. Adv. 6, eaba1425 (2020). DOI: 10.1126/sciadv.aba1425, AAAS. Panel (B) Reproduced from Chen J et al., Int J Biol Macromol, Vol. 272, Pt. 1, 132856, Copyright (2024), with permission from Elsevier. Panel (C) Reproduced from Liang C et al., J Hazard Mater, Vol. 425, 127886, Copyright (2022), with permission from Elsevier. Panel (D) Reproduced from Pan X. et al., Nat Commun 15, 2651 (2024). Open Access CC BY 4.0.

Table 1 reports a selection of functional amyloid fibrils, derived from natural or protein sequences, highlighting their applications as innovative materials. A brief description of a representative example of modified amyloid fibrils for applications in coating, wastewater treatment, and tissue engineering (Figure 4) is reported in the following.

**TABLE 1:** Functional amyloids from natural sequences as innovative materials.

Protein/ Peptide	Category	Application	Ref.
CsgA (*E. coli*)	Biomaterial	Coatings	[78]
β‐lactoglobulin	Biomaterial	Bioremediation	[79, 87]
β‐lactoglobulin	Carrier	Drug delivery	[90, 91]
Ovalbumin	Medicinal	Vaccine adjuvant	[92]
Ovalbumin	Biomaterial	Antimicrobial	[80]
Lysozyme	Sensor	Fluorescence detection	[81]
Lysozyme	Biomaterial	Bioremediation	[82]
Lysozyme	Biomaterial	Antibacterial Drug carrier	[83, 84]
Silk	Biomaterial	Tissue engineering	[85]
Ure2	Biomaterial	Tissue engineering	[86]
Sup35	Biomaterial	Antiviral	[88]

***Coating***. The bacterial protein CsgA secreted from *Escherichia coli* self‐assembles into nanofibers known as Curli fibers, creating robust and biocompatible biofilm [93]. In line with their biological role, these physical properties have inspired their potential use as advanced surface coatings. Indeed, Zhong and coworkers [78] reported modified CsgA proteins that proved to form a versatile coating platform (Figure 4A). Specifically, nanofibrils obtained by genetically engineered CsgA fusion proteins generated thin‐film nanofibers, coating a wide range of substrates (*i.e*., polymers, metals, and complex tridimensional surfaces) while maintaining chemical and mechanical stability. These coatings were further customized to anchor diverse moieties such as nanoparticles or nucleic acids, enabling applications as conductive gold coatings for touch‐sensitive electrodes and pressure sensors or DNA‐functionalized microfluidic channels for bacterial detection [78].

***Wastewater treatment***. The assembly of β‐lactoglobulin and lysozyme into extended fibrillar networks has been exploited to obtain nanomaterials for the removal of organic pollutants [79] and heavy metal ions from water [82, 87]. Zeng group [79] reported an efficient method to use β‐lactoglobulin as a sustainable and reusable material for wastewater treatment (Figure 4B). In detail, the self‐assembling properties of this protein, together with a high number of functional amino acid residues (*e.g*., lysine), were used to fabricate aerogels through a simple three‐step process of glycidyl methacrylate grafting, photo‐crosslinking, and lyophilization. The resulting materials exhibited high porosity and biocompatibility and sufficient mechanical strength to allow absorption of pollutants and consequent separation from water. With a similar aim, Du and coworkers [82] employed lysozyme to develop a platform for selective heavy metal removal (Figure 4C) by transforming water‐soluble lysozyme into robust nanofibers with the aid of polydopamine as a functional adjuvant. The resulting fibers serve as building blocks for the preparation of free‐standing porous membranes *via* vacuum filtration. The membranes exhibit a hierarchical mesoporous architecture with a high surface area, good wettability as well as thermal stability, enabling rapid Pb(II) adsorption through chemisorption mediated by strong complexation with amine and carbonyl groups.

***Tissue engineering***. Among natural proteins, silk fibroin, extracted from silkworm cocoons, is known for its ability to form highly stable β‐sheet structures, which possess remarkable mechanical strength and biocompatibility [94]. For these reasons, silk fibrils turned out to be an ideal material for a variety of applications, including biomedical hydrogels for tissue engineering and regenerative medicine [95]. Recently, Ouyang et al. [85] reported an interesting use of this nanomaterial as a meniscus repair adhesive (Figure 4D). Silk fibroin was chemically modified and combined with an ionic liquid and a growth factor (the protein TGF‐β1) to create a photocurable hydrogel. This multifunctional adhesive not only provided an immediate mechanical bridging and wet adhesion between knee joints but also promotes the tissue regeneration. Overall, silk fibroin acts as a structural and biological template, enabling the formation of a hydrogel that plays a dual function as both a robust adhesive and a regenerative platform.

## 4 Catalytic Amyloids: Expanding the Biocatalysis Toolbox

**FIGURE 5:**
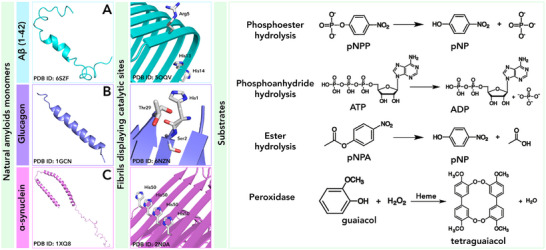
Diagram showing the formation of natural amyloid fibrils such as (A) Aβ42, (B) glucagon, (C) α‐synuclein and their catalytic properties. The monomers assemble into fibrils displaying catalytic sites on their surface. These fibrils exhibit esterase, phosphatase, and oxidation activities.

Looking at the molecular basis of protein misfolding diseases, several authors reported on the ability of amyloids to catalyze a variety of reactions. These findings have suggested a potential involvement of the fibril catalytic role in the onset of the pathological conditions [96]. Indeed, the pathogenic amyloid fibrils, such as those formed by amyloid‐β (Aβ) [97, 98] and glucagon fibrils [99, 100], as well as α‐synuclein [101, 102], are able to catalyze chemical transformations in vitro, demonstrating to be endowed with esterase, phosphatase, and even oxidative activity toward a wide range of substrates (Table 2 and Figure 5).

**TABLE 2:** Selected examples of natural catalytic amyloids and their specific catalytic activities.

Entry	Peptide	Catalytic activity	Substrate	$k_{\mathrm{cat}}$ $\left(10^{-3}s^{-1}\right)$	$k_{\mathrm{cat}}/K_{m}$ $\left(M^{-1}s^{-1}\right)$	Ref.
1	Aβ42	Hydrolysis	pNPA	1.89	0.65	[97]
2	Aβ42	Hydrolysis	AtCh	7.29	0.65	[97]
3	Aβ42	Peroxidase	GU, H_2_O_2_	/	/	[98]
4	Glucagon	Ester hydrolysis	pNPA	2.5	0.57	[99, 100]
5	Glucagon	Phosphoester hydrolysis	pNPP	7	57	[99, 100]
6	Glucagon	Phosphoanhydride hydrolysis	ATP	0.16	2.8	[99, 100]
7	WT α‐synuclein	Ester hydrolysis	pNPA	11	2.9	[102]
8	WT α‐synuclein	Phosphoester hydrolysis	pNPP	0.3	0.6	[102]

In entry 3, the amyloid peptide Aβ42 houses the heme cofactor.

Jelinek and coworkers [97] reported that in the presence of mature β‐amyloid (1‐42) (named Aβ42) fibrils, the model substrate para‐nitrophenyl acetate (pNPA) can be easily hydrolyzed (Table 2 entry 1). Interestingly, addition of metal ions (*e.g*., Zn^2+^, Cu^2+^, and Fe^3+^) to the monomeric peptides did not enhance their catalytic performances. Conversely, divalent ions like Zn^2+^ and Cu^2+^ even led to a reduction in activity. Moreover, Aβ42 can hydrolyze acetylthiocholine (AtCh), a signal of neurodegeneration, and oxidize dopamine and adrenaline, neurotransmitters linked to Alzheimer's disease (Table 2 entry 2). Since the interaction between hemin and amyloids was first investigated for exploring the altered heme metabolism in association with neurodegenerative diseases [103, 104, 105], research has also focused on the binding of β‐amyloid to heme moieties. As reported by Xu and Liu group [98], the Aβ42 fibrils are effectively able to bind heme, with histidine residues (His13 and His14) playing a crucial role in heme coordination. Interestingly, the Aβ‐heme complex catalyzes the oxidation of guaiacol (GU) in the presence of hydrogen peroxide (Table 2 entry 3), with Arg5 likely serving the role of proton source essential for the peroxidase‐like activity.

Hydrolytic activity toward pNPA has been demonstrated also for glucagon amyloid fibrils, unlike their monomeric forms (Table 2 entry 4), as reported by Jelinek and coworkers [99, 100]. Analysis of several glucagon variants allowed relating the observed catalytic activity to the presence of the residues His1, Ser2, and Thr29 in the 29‐residue glucagon peptide, resembling the catalytic triad of hydrolase enzyme active sites [99]. In particular, glucagon fibrils significantly accelerated pNPA hydrolysis, yielding a fourfold increase in reaction rate compared to control experiments. Additionally, the fibrils exhibit enzymatic promiscuity promoting lipid hydrolysis and dephosphorylation, with para‐nitrophenyl palmitate (pNP‐palmitate) and para‐nitrophenyl‐orthophosphate (pNPP) as model substrates. In particular, glucagon amyloid fibrils promote the dephosphorylation of pNPP with high catalytic efficiency, surpassing other amyloid fibrils (Table 2 entry 5). They are also active in the dephosphorylation of a physiological relevant substrate (adenosine triphosphate, ATP) (Table 2, entry 6). Similarly to Aβ42 and glucagon fibrils, wild‐type α‐synuclein amyloid fibers efficiently catalyzed the hydrolysis of pNPA and the dephosphorylation of pNPP with equivalent catalytic efficiencies (Table 2 entries 7 and 8, respectively) [102]. Also for these fibrils, the presence of the histidine residue is essential to promote activity. Indeed, when His at position 50 was substituted with Ala, dephosphorylation activity was notably decreased, while pNPA hydrolysis was unaffected, suggesting the essential role of His50 for phosphatase function but not for esterase activity. All these results uncovered the bright side of amyloid fibrils, emphasizing their potential as enzyme mimics. In the following sections, we describe selected successful examples of artificial catalytic amyloids obtained either by modification of natural amyloid sequences, namely “bioinspired,” or by *de novo* design of self‐assembling peptides. We also illustrate the development of novel catalysts by merging natural or artificial enzymes and amyloid fibrils.

### 4.1 Bioinspired Catalytic Amyloids

The intrinsic catalytic activities exhibited by natural amyloids have stimulated considerable interest in harnessing and modifying natural sequences for the development of tailor‐made catalytic nanomaterials [106, 107, 108, 109, 110, 111, 112, 113, 114, 115, 116]. Moreover, the amyloid ability to bind several metal ions [117, 118, 119] opens up the possibility of mimicking metalloenzymes, thus allowing the catalysis of a variety of reactions beyond hydrolysis, such as oxidations [35]. Here we will first describe catalytic amyloids obtained by introducing cofactor‐independent catalytic sites within bioinspired sequences, then we will move to those that bind a metal cofactor.

***Cofactor‐free bioinspired amyloids***. Lynn and coworkers reached impressive results on unraveling the self‐assembly behaviors of the nucleating core (LVFF) of the Aβ 1–42 peptide of Alzheimer's disease. By studying several variants, they provided a framework to explore how structural adjustments of individual amino acid affect the assembly order and, in turn, the functional properties of the nanomaterial [106]. In an elegant contribution, they demonstrated that the heptapeptide sequence Ac‐KLVFFAL‐NH_2_ (referred as Ac‐KL) is capable of assembling into antiparallel out‐of‐register β‐sheets, which, upon stacking, form nanotubes with a diameter of ∼31 nm (Table 3, entry 1). This arrangement forces N‐terminal lysine residues to be exposed on the nanotube surface. As a consequence, lysine could be mutated with other polar residues, such as Arg (leading to Ac‐RL analogue), without significantly affecting the morphology of the nanotubular assembly. The Ac‐KL nanotubes were capable of promoting the end‐to‐end poly‐imine condensation of 6‐amino‐naphtaldehyde with a 10‐fold higher rate compared to the Ac‐RL analogue, suggesting the main role of the N‐terminal residue in the oligomerization catalysis. Interestingly, the single L7E mutation in the peptide sequence causes the formation of anti‐parallel in‐register β‐strands, altering the supramolecular assembly with variation in the density and exposition of the lysine residues along the nanotubes, which dramatically affects catalysis [106]. These results revealed the plasticity of the Aβ peptide amyloid assemblies in controlling and tuning active site density on the surface of catalytic nanomaterials.

**TABLE 3:** Bioinspired catalytic amyloids and their specific catalytic activities.

Entry	Peptide	Catalytic activity	Cofactor	Substrate	$k_{\mathrm{cat}}$ $\left(s^{-1}\right)$	$k_{\mathrm{cat}}/K_{m}$ $\left(M^{-1}s^{-1}\right)$	Ref.
1	Ac‐KLVFFAL‐NH_2_ (Ac‐KL)	Retro‐ aldolase	/	(±)‐methodol	6.2 × 10^−^^5^	/	[106]
2	Im‐KLVFFAL‐NH_2_ (Im‐KL)	Ester hydrolysis	/	pNP‐oxopentanoate	1.5 × 10^−^^3^	2.1	[107]
				pNP‐pentanoate	1.2 × 10^−^^3^	3.6	[107]
3	Im‐KLVFFAL‐NH_2_ (Im‐KL)	Retro‐ aldolase	/	4‐Hydroxy‐4‐ (6‐methoxy naphthalen‐2‐yl)butanone	4.5 × 10^−^^5^	0.33	[108]
4	Im‐KLVFFAL‐NH_2_ (Im‐KL)	Ester hydrolysis	/	4‐Nitrophenyl‐ 4‐nitro benzoate	1.6 × 10^−^^4^	1.1	[108]
5	Im‐KLVFFAY‐NH_2_ (Im‐KY)	Cascade reaction	/	1‐(6‐methoxynaphthalen‐2‐yl)‐3‐oxobutyl acetate	1.4 × 10^−^^4^	2.8	[108]
6	Im‐KLVFFAY‐NH_2_ (Im‐KY_Cu)	Oxidation	Cu^2+^	4‐chloro‐1‐naphthol	3.3 × 10^−^^4^	0.38	[109]
7	Im‐KLVFFAL‐NH_2_ (Im‐KL_Cu)	RNase	Cu^2+^	2‐Hydroxypropyl‐4‐nitrophenylphosphate	4.4 × 10^−^^4^	0.97	[109]
8	Ac‐HLVFFAL‐NH_2_ (HL)	Cascade reaction	Hemin	MPA	0.13	/	[110]
				GU	0.24	/	[110]
9	Ac‐KLVFFAH‐NH_2_ and Ac‐RLVFFAH‐NH_2_ (KH and RH)	Peroxidase	Hemin	ABTS	0.19	4871	[111]
10	HKLVFFAX‐NH_2_	Asymmetric Michael addition	Cu^2+^	1,2‐unsaturated ketones	/	/	[112]
11	Ac‐NADFDGDQMAVHV‐NH_2_	Phosphoanhydride hydrolysis	Mn^2+^	ATP	3.9×10^−^^6^	5.5×10^−^^8^	[113]
12	Ac‐SDIDVFI‐NH_2_	Phosphoester hydrolysis	Mn^2+^	ATP	11×10^−^^6^	6.9×10^−^^2^	[115]

In the table, ‘Ac’ denotes acetylated N‐terminal, ‘Im’ is referred to Imidazole. In all the cases, the most active peptide analogues are shown. The acronym in parentheses refers to the N and C‐termini residues. In entry 10, ‘X’ refers to all the investigated amino acids.

**FIGURE 6:**
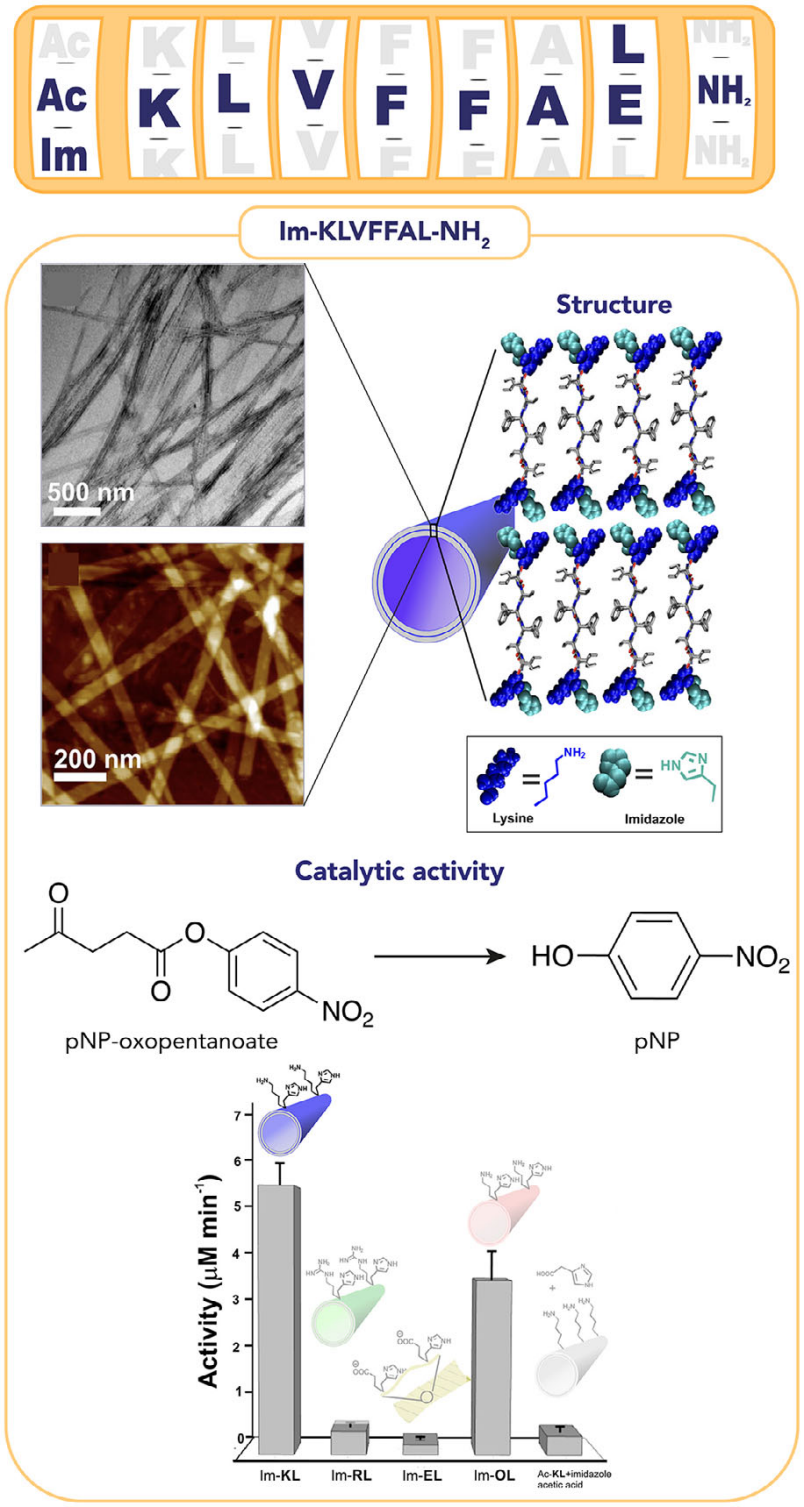
Schematic representation of the Aβ42‐inspired peptides and their catalytic activity. Nanotubes of the Im‐KL peptides facilitate the hydrolysis of inactivated esters through imine formation [107]. Adapted from Sarkhel B. et al., JACS, 2020, 142, 4098–4103. Permission under Copyright 2020, American Chemical Society.

**FIGURE 7:**
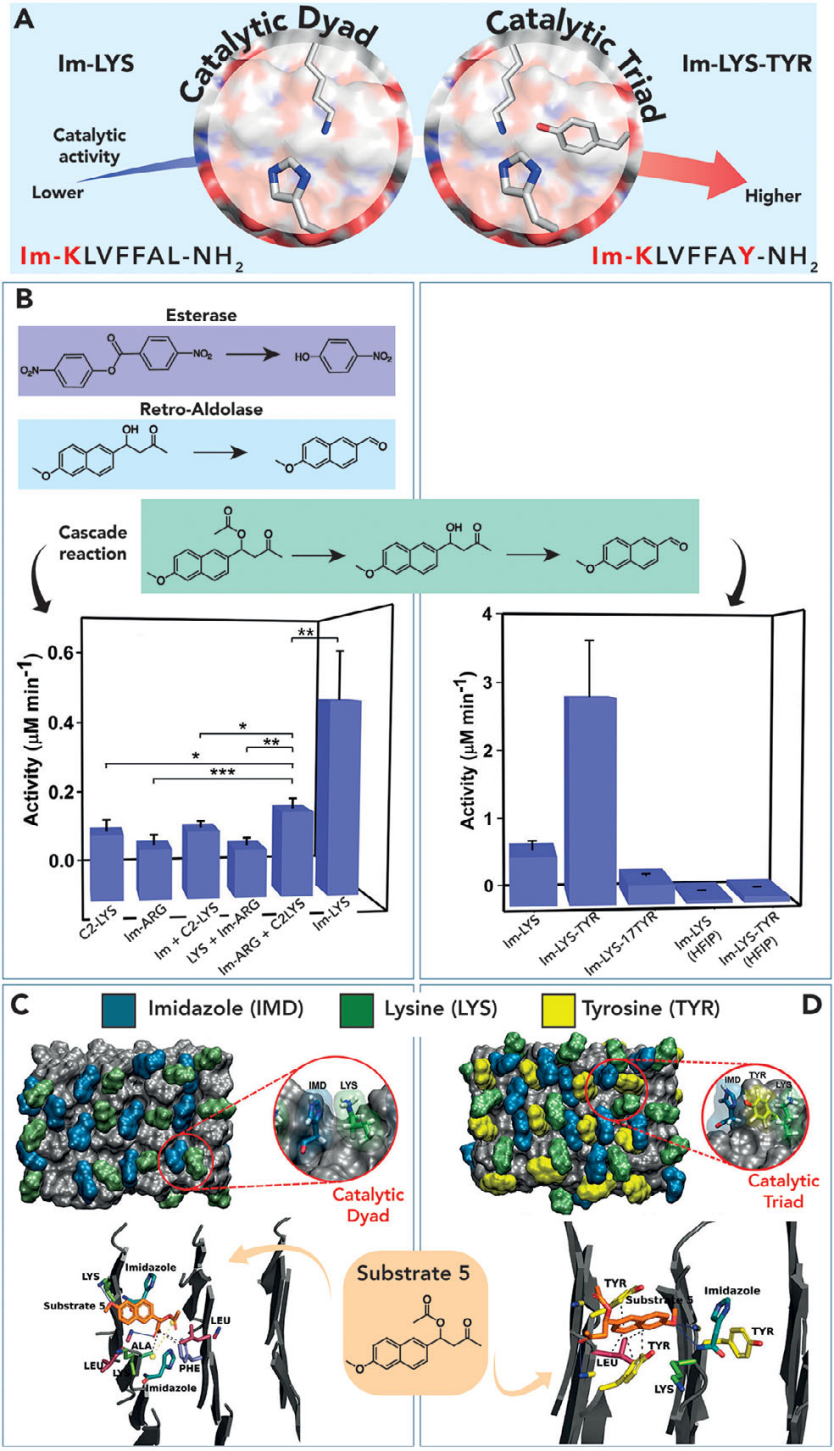
(A) Schematic representation of the catalytic dyad (Imidazole‐Lysine) and triad (Imidazole‐Lysine‐Tyrosine) in the Im‐KL‐based nanotubes and their respective catalytic promiscuity. (B) Catalytic activities of dyad and triad assemblies in cascade reactions. Initial rates are shown for representative systems. (C) Computational models of Im‐LYS and Im‐LYS‐TYR assemblies (Van der Waals surfaces) with substrate docked onto each surface [108]. Adapted from C. Ghosh, et al., Nano Lett. *2023*, 23, 5828–5835. Copyright 2023, American Chemical Society.

Indeed, the KL sequence was subsequently used by Das and coworkers to explore the feasibility of using the amyloid‐like nanotubes for covalent catalysis [107]. To this aim, the N‐terminal amino group of KL was modified with an imidazole (Im) moiety, leading to Im‐KL peptide (Im‐KLVFFAL‐NH_2_, also referred as Im‐LYS, Figure 6, Table 3 entry 2). This sequence efficiently catalyzed the conversion of the keto‐ester p‐nitrophenyl 4‐oxopentanoate (referred as pNP‐oxopentanoate) by Schiff imine formation. The finding that Im‐KL nanotubes exhibited an ∼11‐fold higher hydrolytic activity compared to Im‐RL (also referred as Im‐ARG, Im‐RLVFFAL‐NH_2_) (Figure 6), whereas similar activity was measured for both sequences toward a substrate lacking the keto group, confirmed the effectiveness of the exposed lysine to boost hydrolytic reactivity by covalent catalysis. [107] The versatility of the Im‐KL‐based nanotubes in accommodating more than one catalytic residue (Figure 7A) was further highlighted by comparison with different sequences showing esterase or retro‐aldolase activities (Figure 7) [108, 120].

Im‐KL showed high catalytic efficiency in both the retro‐aldol reaction and ester bond cleavage (Table 3, entries 3 and 4), outperforming sequences containing a single catalytic residue, either imidazole or lysine. Furthermore, the capability of these nanotubes to promote cascade reactions was evaluated by using a dual‐functional substrate, 1‐(6‐methoxynaphthalen‐2‐yl)‐3‐oxobutyl acetate, also referred as substrate 5 (Figure 7B). The highest activity in terms of catalytic efficiency was shown by Im‐KL, with a 48‐fold increase in cascade activity over background, indicating the effectiveness of the catalytic dyad formed by the proximity of lysine and imidazole residues. Moreover, this assembly was able to accommodate a functional catalytic triad resulting from the mutation of the C‐terminal Leu to Tyr, affording Im‐KY (also referred as Im–LYS–TYR, Im‐KLVFFAY‐NH_2_). Nanotubes formed by Im‐KY, exposing the three catalytic residues, displayed a 5.8‐fold increased catalytic efficiency in cascade reaction (Table 3 entry 5 and Figure 7B) compared to Im‐KL. Interestingly, docking experiments of substrate 5 in the active site of either Im‐KY or Im‐KL sequences revealed clear differences in the substrate binding modes. In the Im‐LYS dyad, the substrate preferentially occupies the cross‐β grooves, stabilized by hydrogen bonds of Im and LYS and hydrophobic contacts. This results in a well‐defined but relatively simple binding pocket (Figure 7C). In contrast, the Im–LYS–TYR triad provides a more complex interaction environment. While the substrate binds to same grooves, TYR contributes with additional hydrogen bonds and π‐stacking interactions (Figure 7D), which improve substrate binding affinity.

**FIGURE 8:**
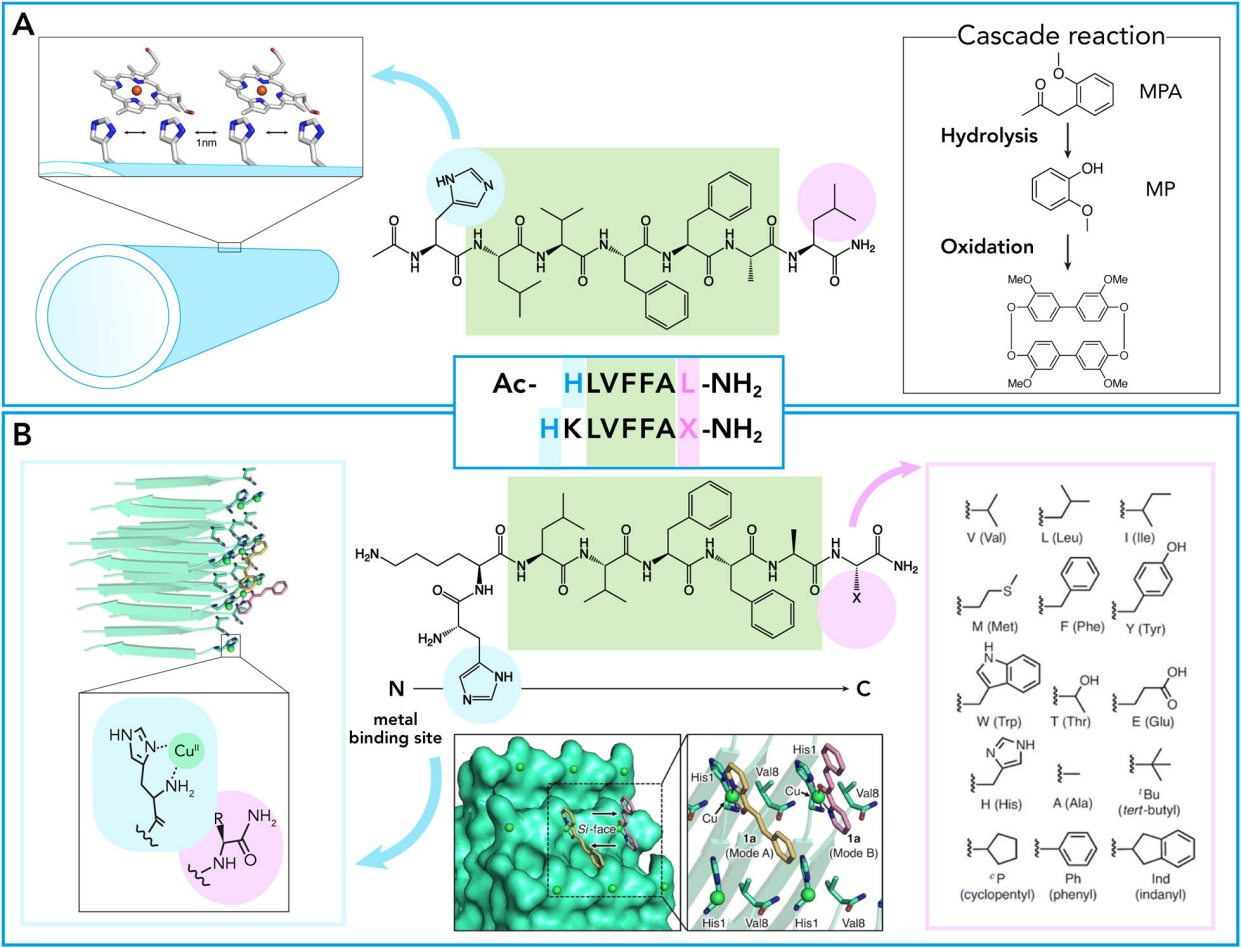
N‐and C‐termini residues modification of Aβ(16–22) and their role in catalysis. (A) Schematic representation of the cascade reaction on the nanotube surface formed by Ac‐HLVFFAL‐NH_2_, along with the chemical structures of amyloid, MPA, MP, and the oxidation product [110]. (B) Amyloid‐copper complex with chemical structures of the side chain R of the amino acid X (C‐terminal position) and molecular model of the self‐assembly peptide H_2_N‐HKLVFFAX‐NH_2_ [112]. (Panel (B) adapted from Fujieda N. et al., *RSC Adv*, 2024, 14, 206. Reproduced with permission from the Royal Society of Chemistry).

***Cofactor‐bound bioinspired amyloids***. The ability of the above mentioned short peptide assemblies to host metal ions was very recently reported. [109] Cu^2+^ binding to the exposed imidazole and phenol moieties of the Im‐KY sequence expanded its catalytic promiscuity toward mimicking the copper binding sites of laccases and galactose oxidases [109]. Fibrils of Cu^2+^ bound Im‐KY (Im‐KY_Cu) were assayed for RNAse and oxidase activity. Interestingly, the catalytic performance was found to be strongly influenced by the pathway chosen for nanomaterial self‐assembly. In detail, two assembly pathways were explored, involving either the addition of Cu^2+^ to the monomeric peptide (pathway‐I), or introduction of Cu^2+^ into preformed assemblies (pathway‐II). Fibrils formed through pathway‐II displayed significantly higher catalytic efficiency compared to those prepared through pathway‐I, both in oxidase activity (Table 3, entry 6) and ribonuclease‐like activity (Table 3, entry 7). Although structural characterization revealed that both assembly procedures provided fibrils, a higher β‐sheet content was found in those produced with pathway‐II compared to pathway‐I, suggesting structural variations that may influence catalytic activity. Further, the potential of the Im‐KY_Cu nanomaterial to promote hydrolase‐oxidase cascade reactions, using 2′,7′‐dichlorodihydrofluorescin diacetate (Ac‐DCFH) as a substrate, was also explored. Notably, Im‐KY_Cu assemblies prepared by pathway II exhibited superior cascade activity not only compared to those prepared by pathway I but also compared to mixtures of natural enzymes (esterase and laccase). This effect was attributed to the presence of arrays of colocalized catalytic units on the surface of Im‐KY_Cu, possibly favoring the channeling of the reaction intermediate through the assembly structure and resulting in higher catalytic activity. Finally, mutation of Lys into His at the N‐terminal of the parent Ac‐KL sequence (Ac‐HLVFFAL‐NH_2_, referred as HL) was aimed at generating a heme‐binding pocket on the surface of the assembled nanotubes (Table 3 entry 8, Figure 8A) [110]. The resulting nanomaterial, named hemin‐HL, displayed peroxidase‐like activity toward GU as a substrate. Further, it was also exploited to promote tandem reactions. In particular, the system was found to efficiently promote the hydrolysis of the 2‐methoxy phenyl acetate (MPA) and the subsequent oxidation of GU using H_2_O_2_. Notably, the system outperforms cytochrome c (Cytc) by 6.8‐fold in the cascade reactivity. By a detailed comparison of the same reaction in the bulk phase, this finding was correlated with intermediate channeling effect likely mediated by the self‐assembled nanotubes.

The advantages of using the short Aβ peptide sequences to construct catalytic nanomaterials with practical applications were recently reported by Yang and coworkers [111]. Mixing of the two sequences Ac‐KLVFFAH‐NH_2_ and Ac‐RLVFFAH‐NH_2_ (namely KH and RH, respectively), with the original KL peptide gave coassembled peptide nanotubes (CA‐PNTs) able to bind hemin. The CA‐PNTs/hemin composites demonstrated higher peroxidase‐like activity in the oxidation of 2,2′‐Azino‐bis(3‐ethylbenzothiazoline‐6‐sulfonic acid) (ABTS) with respect to free hemin, which could be tuned by altering the relative amounts of the short peptides in the coassembly. Among the tested composites, KH‐3/hemin (with a KL:KH ratio of 7:3) exhibited the highest catalytic efficiency in ABTS oxidation, reaching (Table 3, entry 9) approximately 14 times higher than that of the KL/hemin system and 405 times higher than that of free hemin. By integrating this system within an enzymatic reaction cascade, the authors developed a colorimetric method for uric acid (UA) detection that was successfully integrated with smartphone‐based analysis.

The peptides inspired by amyloid core of Aβ42 proven to be very versatile also in reproducing the active sites of more complex metalloenzymes. Fujieda et al. [112] designed new catalytic amyloids with a ‘Histidine Brace’ copper binding site. They developed a library of 12 self‐assembling peptides with a histidine residue at the N‐terminus, whose free amino group and imidazole ring together act as a bidentate ligand for Cu^2+^ ions, and with varied C‐terminal residues (*e.g*., Val, Leu, Ile, Met, Tyr, Trp, etc.) to introduce different steric and electronic environments around the catalytic site. The amyloid fibrils assembly causes the N‐ and C‐terminals to be displayed on the surface, thus behaving as a catalytic center (Figure 8B). Moreover, changing the nature of the C‐terminal residue allows tuning the interaction with substrate through a variety of effects, as π‐π stacking, hydrogen bonds, and electrostatic interactions. These peptides were used to explore selectivity in asymmetric Michael additions between 2‐azachalcone and dimethyl malonate, achieving moderate‐to‐good enantioselectivities and good‐to‐excellent yields (Table 3 entry 10). Notably, aliphatic residues such as Val, Leu, Ile, and Met and some aromatic (Tyr, Trp), proved higher enantioselectivity with respect to more hydrophilic Thr, Glu, and His, demonstrating that the size of the residue is crucial for selectivity. Also, removing or acetylating the N‐terminal histidine drastically reduced enantioselectivity, underscoring its central role in metal coordination and stereoselectivity. All the above‐mentioned examples have highlighted the ability of the Aβ‐derived sequences to promote different catalytic functions.

A different approach was followed by the Diaz‐Espinoza group [113], who developed catalytic amyloids incorporating acidic residues (aspartic and glutamic acid), taking inspiration from the active site of nucleotide‐processing enzymes [113, 114, 115]. By combining the aspartate rich, highly conserved sequence (NADFDGD) of RNA polymerases, involved in the coordination of Mg^2+^ or Mn^2+^ ions, with a fragment with high amyloid propensity (QMAVHV), they obtained a 13‐residue peptide sequence (NADFDGDQMAVHV) able to self‐assemble into amyloids in the presence of Mn^2+^ or Mg^2+^ ions. The Mn^2+^‐bound fibrils catalyzed the hydrolysis of phosphoanhydride bonds of adenosine triphosphate ATP into adenosine mono‐ and diphosphate (AMP and ADP) (Table 3 entry 11). By exploiting the same strategy, another Mn^2+^‐dependent amyloid inspired by the active site of polymerase was developed (SDIDVFI). Also in this case, the self‐assembly process is driven by the presence of the Mn^2+^ ions, as confirmed by transmission electron microscopy (TEM) images that show elongated fibrillar structures. Interestingly, the metal ion is essential not only for initiating aggregation but also for maintaining the amyloid state, as evidenced by experiments using EDTA. These fibrils displayed activity for the hydrolysis of nucleotides (e.g., ATP, GTP, CTP, UTP), a new class of substrates [114]. Recently, the same group [115] demonstrated that for these sequences amyloid assembly and morphology are modulated by the nature of the metal ions (Table 3 entry 12). In the case of Mg^2+^ e Ca^2+^, the fibrils appear more bundled and twisted compared to those formed in the presence of Zn^2+^ and Mn^2+^. MD simulations correlated with experimental observations revealed that manganese and zinc promote more stable amyloid conformations through tight coordination between aspartate residues, resulting in a robust metal‐decorated surface. In contrast, magnesium and calcium destabilize the fibrils. Overall, the described examples demonstrate the possibility of obtaining synthetic self‐assembling peptides by modification of natural sequences, tailored to mimic enzyme‐like catalysis and employed for biotechnological applications [121]. The advantages of using natural peptides, especially for the Aβ‐derived sequences, are strongly linked to their assembly behaviors, that can be tuned by external factors. By strategically positioning combinations of functional groups, researchers have successfully engineered nanotubes and other nanostructures that mimic key features of natural enzyme active sites. These nanomaterials catalyze common reactions such as ester cleavage but also enable complex cascade processes and peroxidase‐like activities. Nevertheless, several limitations remain. Indeed, this strategy inherently constrains the structural diversity of achievable nanostructures, as it relies on sequence fragments and folding principles derived from natural proteins, thereby excluding the exploration of self‐assembling sequences that are unrelated to natural ones. *De novo* design of catalytic amyloids, discussed in the next section, seeks to overcome these limitations by greatly expanding the accessible sequence space and demonstrating that complex, functional architectures can emerge even from highly minimalist, non‐natural sequences.

### 4.2 De Novo Catalytic Amyloids

With the aim of further expanding the field of catalytic amyloids, different catalytic sites have been engineered into *de novo* self‐assembling peptide sequences [22, 34]. In contrast to using naturally derived sequences, *de novo* design avoids evolutionary constraints, enabling the engineering of tunable and highly specialized catalytic systems. Designing *de novo* peptide sequences offers the advantage of precise control over structure and functionality, since sequences can be tailored to optimize self‐assembly and arrangement of catalytic motifs.

Nevertheless, differently from natural sequences for which the aggregation properties are mostly known in detail, for *de novo* designed peptides it is necessary to analyze many sequences to select the best performing in terms of assembly and catalytic behaviors. The selected examples reported in this section and in Table 4 [122, 123, 124, 125, 126, 127, 128, 129, 130, 131, 132, 133, 134, 135, 136, 137, 138, 139, 140, 141, 142, 143, 144, 145, 146, 147] will provide an overview of the construction of catalytic amyloids from the ground up. First, we will describe prominent examples of cofactor‐free catalytic amyloids and then those that require binding of a metal cofactor. The common thread for all the systems described is that the self‐assembly of even single residues or small dipeptides into amyloid fibrils may provide complex architectures with the functional diversity, efficiency, and flexibility needed for catalysis.

**TABLE 4:** Metal‐binding or cofactor free *de novo* designed catalytic amyloids and their specific catalytic activities.

Entry	Peptide	Catalytic activity	Cofactor	Substrate	$k_{\mathrm{cat}}$ $\left(s^{-1}\right)$	$k_{\mathrm{cat}}/K_{m}$ $\left(M^{-1}s^{-1}\right)$	Ref.
1	PepNTs‐His‐Arg_max_	Ester hydrolysis	/	pNPA	1.4×10^−^^3^	1.8	[122]
2	CoA‐HSD_max_	Ester hydrolysis^a^	/	pNPA	3×10^−^^3^	0.18	[123]
3	Lauryl‐VVAG‐D/H/S	Ester hydrolysis^b^	/	pNPA	4.4×10^−^^3^	127	[124]
4	E/S/H	Ester hydrolysis^c^	/	pNPA	8×10^−^^5^	0.19	[125]
5	Q11HR_max_	Ester hydrolysis	/	pNPA	2.6×10^−^^3^	0.15	[126]
6	H5‐SG4‐Q11	Oxidation^d^	/	TMB	1×10^−^^3^	2.9	[127]
7	VK2H	Ester hydrolysis	/	pNPA	7×10^−^^2^	19	[128]
8	Ac‐HYHYHYHYH‐ NH_2_	Ester hydrolysis	/	pNPA	3.5×10^−^^3^	1.6	[129]
9	Ac‐IHIHIQI‐NH_2_	Ester hydrolysis^e^	Zn^2+^	pNPA	/	360	[130]
10	Ac‐LHLHLRL‐NH_2_	Ester hydrolysis	Zn^2+^	pNPA	2.3×10^−^^2^	15.7	[131]
11	Ac‐IHIHIQI‐NH_2_	Ester hydrolysis^f^	Zn^2+^	pNPA	0.24	450	[132, 133]
12	Ac‐IHIHIQI‐NH_2_	Ester hydrolysis	Zn^2+^	Z‐L‐Phe‐ONp	0.11	2×10^4^	[134]
13	Ac‐IHIHIQI‐NH_2_	Ester hydrolysis	Zn^2+^	Boc‐L‐Asn‐ONp	1.8×10^−^^2^	3×10^3^	[134]
14	Ac‐IHIHIQI‐NH_2_	Oxidation	Cu^2+^	DMP, O_2_	6.6×10^−^^3^	31	[135]
15	FHFHFdopaF	Oxidation	Cu^2+^	L‐DOPA	13×10^−^^4^	22	[136]
16	FHFHFdopaF	Oxidation	Cu^2+^	Epinephrine	19×10^−^^4^	42	[136]
17	Ac‐IHIHIYI‐NH_2_	Ester hydrolysis	Zn^2+^	pNPA	8.3×10^−^^3^	355	[137]
18	Ac‐IHIHIYI‐NH_2_	Phosphoester hydrolysis	Cu^2+^	Paraoxon	8×10^−^^5^	3×10^−^^2^	[138]
19	Ac‐IHIHIYI‐NH_2_	Hydrolysis/ Oxidation	Cu^2+^	DCFH	/	4.8	[138]
20	Ac‐IHVHLQI‐NH_2_	Ester hydrolysis	Zn^2+^	pNPB	1.76	128	[139]
21	GYGYGYG	Ester hydrolysis	Cu^2+^	pNPA	5.2×10^−^^3^	3.8	[140]
22	GYGYGYG	Ester hydrolysis	Zn^2+^	pNPA	6.4×10^−^^3^	6.0	[140]
23	Phe	Ester hydrolysis	Zn^2+^	pNPA	/	76.5	[141]
24	Phe	CO_2_ hydration	Zn^2+^	CO_2_	7.8	962	[141]
25	Phe	Oxidation	Cu^2+^	2,4‐DP	11.9	6.3×10^−^^2^	[142]
26	Fmoc‐Lys/Phe and His	Oxidation	Hemin	Pyrogallol	17.4	/	[143]
27	Ac‐LILHLFL‐NH_2_	Cyclo‐propanation^d^	Hemin	Styrene	0.90	326	[144]
28	Ac‐LMLHLFL‐NH_2_	Oxidation^g^	Hemin	TMB	0.47	4.7×10^4^	[145]
29	Ac‐LMLHLFL‐NH_2_	Oxidation	Hemin	ABTS	2.4	3.0×10^5^	[145]
30	Ac‐LMLHLFL‐NH_2_	Oxidation^h^	Hemin	oDP	1.9×10^−^^4^	0.45	[145]
31	Ac‐LMLHLFL‐NH_2_	Oxidation^i^	Hemin	^I^TMB	6×10^3^	13×10^4^	[146]
32	c16‐MHL_3_K_3_‐CO_2_H	Oxidation	Hemin	TMB	18.5×10^−^^2^	21.5	[147]

Parameters in the table obtained at:

^a^ pH 9.4,

^b^ 250 µM,

^c^ pH 8,

^d^ pH 6,

^e^ pH 10.3,

^f^ 200 MPa and 38°C,

^g^ pH 6.5,

^h^ in toluene,

^i^ ‘I’ is from the electrochemical measurements.

***Cofactor‐free de novo catalytic amyloids***. The discovery that the core FF motif of the Alzheimer β‐amyloid peptide forms nanotubes in solution [148] stimulated its applications as a minimal peptide sequence for engineering catalytic activity. Considering the role of histidine in the catalytic mechanism of native hydrolases, Liu and coworkers [122] reported an imidazolyl‐containing amphiphilic tripeptide (Fmoc‐FFH‐CONH_2_) with the aim of constructing an artificial hydrolase. This tripeptide self‐assembles into nanotubes, catalyzing the hydrolysis of pNPA substrate. Moreover, coassembled structures were obtained by mixing the Fmoc‐FFH‐CONH_2_ peptide with an analogue bearing Arg in place of His (Fmoc‐FFR‐CONH_2_), with the aim of incorporating guanidyl groups into the nanotubes, serving as binding sites for carbonyls. The catalytic performances of the nanomaterial were enhanced through the optimization of the molar ratio between catalytic (Fmoc‐FFH‐CONH_2_) and binding sites (Fmoc‐FFR‐CONH_2_), and the best coassembled peptide nanotubes (PepNTs‐His‐Arg_max_, with a ratio of 20:1 of Fmoc‐FFH‐CONH_2_:Fmoc‐FFR‐CONH_2_) demonstrated a catalytic efficiency (Table 4, entry 1) increase of 519‐fold compared to a non‐catalytic system. The proposed mechanism involves the stabilization of a transition state through imidazolyl and guanidyl groups, which promotes efficient hydrolysis [149, 150]. Additional structural features were integrated into short peptide‐based amyloid assemblies to mimic the active site of natural hydrolases. In this respect, several de novo nanosystems were engineered to contain a catalytic triad composed of Ser, His, and Asp [123, 124] or Ser, His, and Glu [125].

The simplest among these systems was reported by the Qi group [123], that implanted the catalytic triad into minimal tripeptide self‐assembling sequences. The triad residues (His, Ser, Asp) were conjugated at the C‐terminus of the Fmoc‐FF common dipeptide unit. Through the coassembly of these three peptides in various ratios (*e.g*., Fmoc‐FFH/Fmoc‐FFS/Fmoc‐FFD, 1:1:1 referred as CoA‐HSD or 40:1:1 referred as CoA‐HSD_max_), different nanostructures were obtained. The hydrolytic activity was assessed using pNPA as a substrate, and the CoA‐HSD_max_ resulted to be more active with respect to both CoA‐HSD and to the assembly of the single Fmoc‐FFH peptide (SA‐H), showing a catalytic efficiency of $k_{\mathrm{cat}}/K_{m}=0.18\;M^{-1}s^{-1}$ (Table 4 entry 2). It is worth noting that the catalytic efficiency of CoA‐HSD_max_ is approximately 10‐fold lower than that of the previously described PepNTs‐His‐Arg_max_, despite the former exhibiting a twofold higher k_cat_ value. While the presence of the catalytic triad (HSD) effectively speeds up catalytic turnover, the Arg sidechains in the PepNTs‐His‐Arg_max_ nanotubes markedly improve catalytic efficiency by increasing substrate affinity (lowering the K_m_).

Guler and coworkers [124] adopted a similar approach and constructed nanostructures in which each amino acid of the catalytic triad (D/H/S) was derived from a different self‐assembling amphiphilic peptide (Lauryl‐VVAG‐X, with X is referred to D, H, or S). Additional complexity was introduced in the sequence with respect to the Fmoc‐FF scaffold, featuring a N‐terminal lauryl group and a tetrapeptide core, with the aim of creating hydrophobic catalytic pockets resembling those found in natural enzymes. The catalytic activity was evaluated using both pNPA and acetylcholine, a natural substrate of the enzyme acetylcholinesterase. Kinetic analyses demonstrated that the ternary assembly of Lauryl‐VVAG‐X peptides holding all the D/H/S residues is more efficient (Table 4, entry 3) compared to the CoA‐HSD_max_ assembly ($k_{\mathrm{cat}}/K_{m}=127\;M^{-1}s^{-1}$; Table 4, entry 2) highlighting the importance of incorporating hydrophobic patches into the nanomaterial. Indeed, the ternary Lauryl‐VVAG‐X fibers exhibit a substantially lower K_m_ compared to the previously reported examples, indicating an enhanced ability to bind substrates in a manner reminiscent of natural enzymes. In a more recent study, Rapaport and coworkers [125] have integrated the catalytic triad E/H/S along amphiphilic β‐sheet peptides, with a focus on exploring how the order of catalytic triad residues along β‐strands influences peptide assembly and catalytic activity. Three peptides were designed with the common sequence Ac‐Cys‐Phe‐X‐Phe‐Y‐Phe‐Z‐Phe‐Pro‐NH_2_, where X, Y, and Z represent the triad residues (Glu, His, Ser) in different orders. Their catalytic performances were evaluated in the hydrolysis of pNPA, with Ac‐Cys‐Phe‐Glu‐Phe‐Ser‐Phe‐His‐Phe‐Pro‐NH_2_ (ESH) showing the highest catalytic efficiency ($k_{\mathrm{cat}}/K_{m}=0.19\;M^{-1}s^{-1}$; Table 4 entry 4) among the analogues. Structural investigations by Cryo‐EM, Fourier transform infrared spectroscopy (FTIR), and Grazing Incidence X‐ray Diffraction (GIXD) revealed that the activity of the nanostructures was strongly dependent on their supramolecular organization. Despite all peptides formed antiparallel β‐sheet structures, ESH was less prone to elongation than the others, reaching the proper balance between structural order and chain flexibility, which may be crucial for catalysis. Further, the electrostatic interactions among catalytic residues also played a critical role in assembly and activity. In particular, the positioning of the serine close to three histidine residues in the ESH peptide assembly likely contributed to its higher catalytic performance.

Another notable example of a cofactor‐free catalytic amyloid was given by Zhang and coworkers [126], who developed two peptides (HSGQQKFQFQFEQQ‐NH_2_ named Q11H and RSGQQKFQFQFEQQ‐NH_2_ named Q11R), capable of forming coassembled nanofibers and catalyzing ester hydrolysis (Table 4 entry 5). This behavior is not exclusive to these sequences but extends to other glutamine‐rich peptides. Indeed, glutamine is known for its strong amyloid‐forming propensity thanks to the hydrogen‐bonding ability of its polar side chain, which can both stabilize the amyloid structure and contribute to catalytic function. As described in previous examples, the His residue in Q11H was introduced to promote ester hydrolysis, whereas the Arg residue in Q11R was incorporated to facilitate substrate binding. Coassembled fibrils formed at an optimized Q11R:Q11H ratio (1:10, named Q11HR_max_) exhibited a catalytic efficiency (Table 4 entry 5) comparable to those of other minimalist peptide assemblies containing hydrolase catalytic triad residues (Table 4, entries 2–4). In further research, Wang group [127] linked a His‐rich pentapeptide (NH_2_‐HHHHH‐COOH, named H5) with the fibril‐forming Q11 peptide (NH_2_‐QQKFQFQFEQQ‐CONH_2_) in a single sequence, with spacers composed of serine and glycine residues (H5‐SGn‐Q11, with n indicating the number of spacer residues). The H5 peptide can catalyze TMB oxidation upon activation of H_2_O_2_, similarly to heme peroxidases, but in the absence of a metal cofactor. Although H5‐Q11 fibrils display peroxidase‐like activity, the introduction of a flexible peptide spacer (SG4) optimized histidine distribution and minimized structural stress. Enzyme kinetics analysis revealed that H5‐SG4‐Q11 exhibited significantly higher catalytic efficiency than H5 alone (Table 4 entry 6), although it remains lower than that of natural heme enzymes.

Ideally, as in natural enzymes, the catalytic activity of peptide nanostructures should be reversibly regulated. In enzymes, such control is achieved through physicochemical stimuli, including binding of ligands, light irradiation, temperature or pH changes. Replicating this type of stimuli‐responsive behavior in artificial systems remains a major challenge, as it requires integrating responsiveness with the precise arrangement of catalytic groups into simple peptide sequences. To this purpose, different research groups have explored the design of artificial switchable enzymes based on amphiphilic self‐assembling peptides. The pH‐switchable artificial hydrolase named VKH2 was designed by introducing a catalytic histidine into the sequence of a pH‐responsive β‐hairpin peptide named MAX1 [128]. In particular, MAX1 consists of two β‐strands made of repeated valine‐lysine (VK) units, connected via a ^D^PPT turn sequence. In VK2H, the N‐terminus was modified by adding an HSG catalytic sequence. Increasing the pH triggers the assembly of VKH2 into fibrils, which in turn form a stable hydrogel. This process is driven by lysine side chain deprotonation, enabling hydrogen bonding between the peptide strands. This pH‐driven assembly allows the formation of a structured catalytic microenvironment, where the hydrophobic valines create a substrate‐binding site within the fibrils. The catalytic activity of VK2H was assessed using pNPA as a substrate, displaying hydrolytic activity at pH 9.5 (Table 4 entry 7). Both the self‐assembly of fibrils and their hydrolytic activity are pH responsive and reversible, with a decrease in pH causing a switch into the random coil, inactive form of the peptide. Reversibility studies demonstrated that VK2H retained its switchable enzymatic function over multiple pH cycles as well as its hydrogel, enabling a phase‐dependent reaction mechanism, useful for applications that require controlled bio‐transformations. Moreover, Ventura and coworkers [129] developed a set of *de novo* short peptides (HY7, HY8, and HY9) composed of an alternate pattern of His and Tyr residues. In addition to display a pH‐responsive behavior, these amyloid‐like nanofibers have a dual catalytic function: the presence of His provides HY fibrils with promote hydrolytic activity, (Table 4, entry 8), while Tyr enables oxidative electrocatalysis, both in the absence of any external cofactor. Although the catalytic efficiencies are still far from those of natural biocatalysts, the activity of HY peptides is comparable to those of other non‐metal‐dependent amyloid‐based hydrolases [126].

**FIGURE 9:**
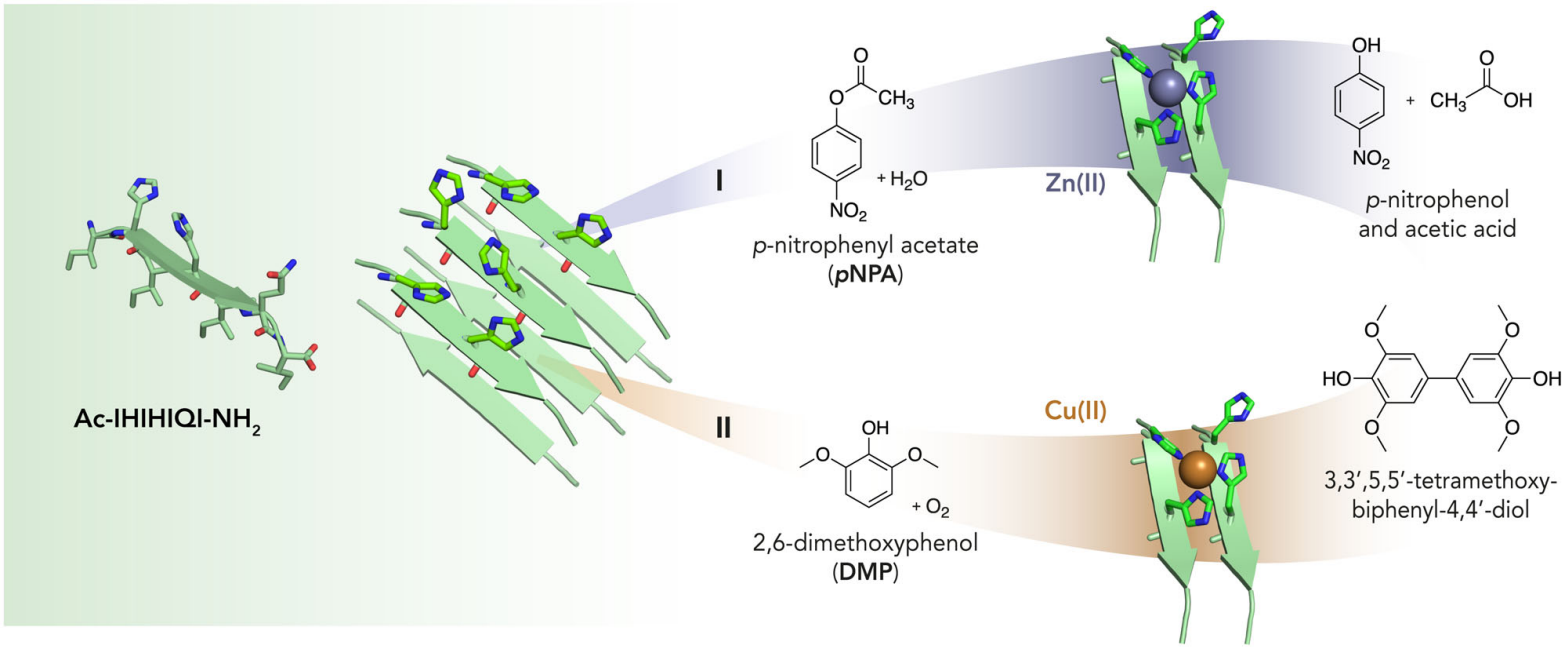
The *de novo* β‐strand peptide Ac‐IHIHIQI‐NH_2_ binds metal ions, promoting catalytic activity. Upon binding zinc (I), it facilitates the hydrolysis of pNPA, and upon binding copper (II), it catalyzes the oxidation of DMP by activating dioxygen [152]. Reprinted from Leone L. et al., J. Pept. Sci., 2024, 30, e3606, with permission of John Wiley and Sons, Copyright 2024 European Peptide Society and John Wiley and Sons Ltd.

***Cofactor‐bound de novo catalytic amyloids***. Beyond the investigation of catalytic amyloids with cofactor‐independent active sites, considerable efforts have also been devoted to the incorporation of metal‐binding motifs into amyloid fibrils. Among them, zinc‐binding sites have been privileged targets, reflecting the abundance of zinc centers in numerous hydrolases and carbonic anhydrases (CAs). In this context, pioneering work from De Grado and Korendovych [130] focused on engineering a Zn^2+^ site into designed amyloid sequences, affording functional hydrolytic nanomaterials. Inspired by the active site of Carbonic Anhydrase (CA) and starting from the β‐sheet heptapeptide LKLKLKL sequence, the authors generated a library of *de novo* designed self‐assembling peptide mutants, able to perform esterase activity (Figure 9). Several modifications to the heptapeptide were strategically designed to retain its hydrophobic periodicity, a critical feature for maintaining structural stability (*i.e*., Ac‐IHIHIQI‐NH_2_), while introducing a functional Zn^2+^ binding site. In particular, the leucine residues were mutated to other apolar residues (*i.e*., Ile), while Lys residues were substituted with other polar residues. Specifically, two His residues at positions 2 and 4 created a binding site for Zn^2+^ ion, enabling metal coordination and catalysis. Various residues with differing pKa values, including acidic (Asp, Glu), neutral (Gln, Tyr), and basic (His, Lys, Arg) residues, were tested at position 6 to further refine the peptide functionalities [130]. Among the synthesized peptides, the analogue with Ile residues substituting Leu and containing Gln at position 6 (Ac‐IHIHIQI‐NH_2_, named peptide 7IQ) exhibited the highest catalytic efficiency in the hydrolysis of pNPA ($k_{\mathrm{cat}}/K_{m}=360\pm 30\;M^{-1}s^{-1},\;\mathrm{pH}\;=10.3$; Table 4, entry 9). Remarkably, the outstanding catalytic performance of these short peptides makes them competitive with the natural enzyme, as reported for CA [151].

In order to understand the relationship between β‐sheet arrangement of the peptides and catalytic activity, molecular dynamics [153, 154] XRD [137] and solid‐state NMR of the Ac‐IHVHLQI‐NH2 analogue [155] provided crucial insights. The enhanced catalytic activity of 7IQ peptide was attributed to the twisted parallel β‐sheet structure adopted in the fibrillar form, exhibiting balanced rigidity and flexibility, which is better at mimicking the active site of the enzyme. In contrast, the less active analogue with Arg at position 6 forms planar antiparallel β‐sheet, which is more rigid and therefore less effective in catalysis [155]. More recently, Heerde and coworkers reported the cryo‐EM structure of fibrils formed by the Ac‐LHLHLRL‐NH_2_ sequence (for pNPA hydrolysis) (Table 4 entry 10), highlighting subtle structural differences with respect to more reactive analogues [131]. In particular, cryo‐EM analysis of fibrils formed by Ac‐LHLHLRL‐NH2 peptide showed multiple morphologies sharing a common architecture. The fibrils consisted of protofilaments with two cross‐β sheets paired in a zipper‐like manner via hydrophobic leucine interactions. The backbone conformation Ac‐LHLHLRL‐NH_2_ was similar to that observed in the solid‐state NMR structure of Ac‐IHVHLQI‐NH_2_. However, the latter involved the presence of two conformations differing for the histidine rotamers and alternating in consecutive layers of the fibril. These conformers were not found in the structure of Ac‐LHLHLRL‐NH_2,_ where interactions between peptides in the fibril core and those in the outer leaflet both participated in zinc binding site. A key issue with this arrangement is the limited solvent exposure of the catalytic center, which could hinder substrate access, thus affecting catalysis.

A major challenge in using amyloid‐based nanomaterials in technical applications is their unclear stability and catalytic performance under industrially relevant conditions. To this aim, Winter and coworkers [132, 133] investigated both the robustness and catalytic performances of the Zn^2+^‐bound fibrils formed by peptide 7IQ under high hydrostatic pressure and temperature. First, these fibrils demonstrated no significant secondary structural changes when exposed to a wide range of pressures (0.1–400 MPa) and temperatures (20–60°C), as confirmed by FTIR spectroscopy. Furthermore, not only was the catalytic efficiency in the hydrolysis of pNPA retained, but also it was significantly enhanced under high pressure and temperature (at 200 MPa and 38°C, Table 4 entry 11). Their stability and enhanced catalytic efficiency under harsh conditions make them ideal candidates for applications in flow reactors and filtration systems. Moreover, Koksch and coworkers [134] investigated the stereoselective hydrolysis of nitrophenyl esters using Zn^2+^‐bound 7IQ fibrils. This study revealed that by using N‐benzyloxycarbonyl (Z) protected L‐phenylalanine (Z‐L‐Phe‐ONp) or N‐terminal Boc protected L‐asparagine p‐nitrophenyl esters (Boc‐L‐Asn‐ONp) as chiral substrates (Table 4 entry 12 and 13, respectively), the catalytic efficiency was notably higher with the more hydrophobic Phe compared to Asn as a substrate. In terms of enantioselectivity, Zn^2+^‐bound 7IQ fibrils showed a clear preference for the L‐enantiomer of both Z‐Phe‐ONp and Boc‐Asn‐ONp, further suggesting that the peptide catalytic site is finely tuned to recognize the stereochemistry of its substrates. To broaden the array of catalytic reactions and substrates, further modifications were made to the 7IQ sequence or in the nature of the metal cofactor. Regarding the choice of the metal ion, Korendovych and coworkers [135] replaced Zn^2+^ with Cu^2+^ ions into 7IQ fibrils, developing a catalytic amyloid able to oxidize 2,6‐dimethoxyphenol (DMP) in the presence of O_2_ (Table 4, entry 14) [135]. As in the case of Zn^2+^, the His‐X‐His motif in the sequence was identified as optimal for copper coordination and catalytic function. The coordination properties of copper bound to the self‐assembling peptide were investigated by EPR spectroscopy, which revealed a type 2 copper coordination environment. With this work, authors proved that short copper‐binding peptides could act as catalysts for oxygen activation.

More recently, Korendovych group [136] expanded the reactivity of Cu^2+^‐bound self‐assembled peptides to the oxidation of catecholamine neurotransmitters such as L‐DOPA, epinephrine (EP), norepinephrine, dopamine, and serotonin in the presence of O_2_ or H_2_O_2_. The phenylalanine‐rich assembly Cu‐FHFHFYF displayed the highest catalytic efficiencies, reaching $k_{\mathrm{cat}}/K_{m}=8.7\;M^{-1}s^{-1}$ and $k_{\mathrm{cat}}/K_{m}=38\;M^{-1}s^{-1}$ for L‐DOPA and epinephrine, respectively, in the presence of H_2_O_2_. The activity was further improved by introducing L‐DOPA in the peptide sequence (FHFHFdopaF), achieving $k_{\mathrm{cat}}/K_{m}=22\;M^{-1}s^{-1}$ for L‐DOPA (Table 4 entry 15) and $k_{\mathrm{cat}}/K_{m}=42\;M^{-1}s^{-1}$ for epinephrine (Table 4 entry 16). These values place catalytic amyloids within two orders of magnitude of natural tyrosinases, indicating that short peptide fibrils can act as efficient abiotic oxidase catalysts. Oxidation of these substrates also produces melanin, whose morphology was found depending on the peptide sequence, indicating a dual catalytic and templating function of the fibrils. The FHFH motif (QFHFHWG) was also identified in the Zn^2+^‐binding β‐sheet region of human carbonic anhydrase VII (HCA VII), suggesting a role of HCA VII in neurotransmitter degradation relevant to neurodegeneration. Interestingly, Cryo‐EM structures of QFHFHWG fibrils revealed an unexpected level of architectural complexity for a short peptide assembly. Two polymorphs, resolved at ∼3.5 Å and ∼5.2 Å, shared a C2‐symmetric dimeric core differing in their outer layers, deviating from canonical cross‐β motifs.

On a different side, the effects of peptide length and sequence on the structure and catalytic properties of the assembly were investigated by Serpell and coworkers [137]. The authors reported a library of six amphiphilic analogues of 7IQ (7–11 residues), based on the same alternating isoleucine/histidine motif as 7IQ, but replacing the Gln at position 6 with a Tyr. Peptide analogues with C‐ and/or N‐terminal capping and longer sequences were also included to examine the effect of multiple metal binding sites on the nanofibrils surface. Biophysical analyses (CD, FTIR, XRD and TEM) demonstrated that all peptides formed amyloid fibrils both in the presence and in the absence of Zn^2+^ ions. However, assays of pNPA hydrolysis revealed no catalytic activity for peptides with free N‐ and C‐terminals even in the presence of Zn^2+^, suggesting that terminal blocking is crucial for function. This finding suggests that acetylation and amidation not only stabilize the supramolecular architecture but also play a role in shaping the active site. In fact, the free termini may introduce unfavorable electrostatic repulsions or disrupt the histidine arrangement required for Zn^2+^ coordination. In contrast, peptides with capped‐ends exhibited enzymatic activity. Notably, Ac‐IHIHIYI‐NH_2_ peptide named 7IY, emerged as the most efficient catalyst, exhibiting a catalytic efficiency $(k_{\mathrm{cat}}/K_{m}=355\;M^{-1}s^{-1}$; Table 4 entry 17) comparable to that of the previously reported 7IQ (Table 4 entry 9). Interestingly, this almost unchanged efficiency arises from a nearly 10‐fold reduction in both k_cat_ and K_m_, suggesting that the Tyr residue contributes primarily to enhancing substrate affinity. Subsequently, Korendovych group [138] reported that the same 7IY peptide were able to bind also Cu^2+^ ions, forming amyloid‐like fibrils. Such Cu^2+−^bound fibrilscatalyze the hydrolysis of the toxic organophosphate pesticide paraoxon (Table 4 entry 18), moderate catalytic efficiency ($k_{\mathrm{cat}}/K_{m}=3\cdot 10^{-2}\;M^{-1}s^{-1}$). The hydrolysis of this substrate remains a challenge due to its low reaction rate, resulting in prolonged persistence in the environment. For this reason, 7IY catalytic fibrils were also tested after deposition onto a 0.22 µm PES filter membrane, providing a continuous flow system for water detoxification. Moreover, 7IY fibrils are able to promote a reaction cascade involving the hydrolysis of 2′,7′‐dichlorofluorescin diacetate (DCFH‐DA) to 2′,7′‐dichlorofluorescin (DCFH) and its subsequent oxidation to 2′,7′‐dichlorofluorescein (DCF) (Table 4 entry 19) [138].

Lou and coworkers [139] also tested additional modifications to the heptapeptide sequence of 7IQ, targeting the hydrolysis of complex substrates besides pNPA. A new analogue of 7IQ containing Val in position 3 and Leu in position 5 (Ac‐IHVHLQI‐NH_2_) was tested, along with 7IQ and 7IY, for hydrolytic activity in the presence of Zn^2+^ ions. These fibrils were able to hydrolyze a variety of substrates, including structurally complex compounds such as p‐nitrophenyl butyrate (pNPB), p‐nitrophenyl octanoate (pNPO) and p‐nitrobenzyl salicylate (pNPS) with high catalytic efficiency (for pNPB, $k_{\mathrm{cat}}/K_{m}=128\;M^{-1}s^{-1}$; Table 4 entry 20). Additionally, these fibrils were also tested in the degradation of a widely used plasticizer, di(2‐ethylhexyl)phthalate (DEHP), to assess their potential for environmental applications. The same sequence was found to be the most active also in the degradation of DEHP, showing a degradation percentage of 87% in 60 min. TEM analysis revealed that Zn^2+^‐Ac‐IHVHLQI‐NH_2_ formed a denser fibrillar network compared to the other peptides. This structural feature may underlie its superior catalytic performance, likely by promoting more favorable substrate interactions within the fibrillar environment.

**FIGURE 10:**
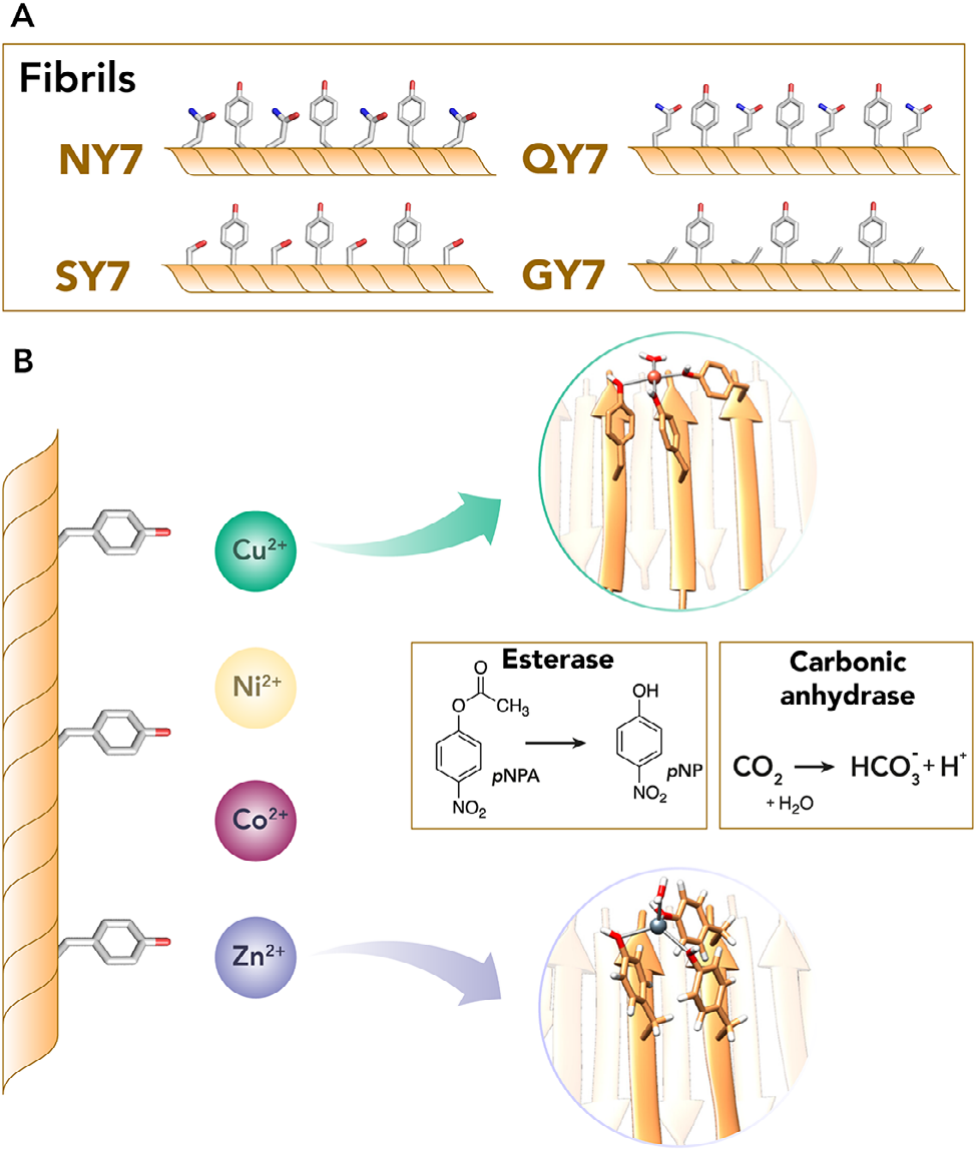
Schematic representation of the strategy adopted for the construction of (A) Tyr‐enriched peptides. (B) Tyr residues binding divalent metal ions for esterase and carbonic anhydrase activity [140]. Adapted from Navarro S. et al., *ACS Nano* 2023, 17, 16968–16979. Reproduced with permission of American Chemical Society, Copyright 2023 licensed under CC‐BY 4.0.

Besides the use of histidine‐rich amphiphilic peptides, Navarro et al. explored the coordination of Cu^2+^, Co^2+^, Ni^2+^, and Zn^2+^ to prion‐inspired amyloid fibrils composed of Tyr‐enriched peptides (NYNYNYN as NY7, QYQYQYQ as QY7, SYSYSYS as SY7 and GYGYGYG as GY7) [140]. Indeed, the phenolate oxygen of the exposed Tyr residues could form stable interactions with metal ions, allowing esterase activity in the hydrolysis of pNPA. In particular, GY7 fibrils showed the highest efficiency among the analogues in both Cu^2+^ and Zn^2+^‐bound forms (Table 4 entries 21 and 22, Figure 10), although their catalytic efficiencies remain approximately two orders of magnitude lower than those of the 7IQ and 7IY peptides.

Moreover, these fibrils were also tested for carbonic anhydrase activity, which involves the reversible hydration of CO_2_ to bicarbonate. Notably, some fibrils exhibited CA activity approaching that of the natural enzyme in the same conditions. Among the library of Zn^2+^‐coordinated amyloid‐like assemblies that mimic carbonic anhydrases, Gazit and coworkers [141] demonstrated the ability of a single amino acid, specifically phenylalanine (Phe), to self‐assemble into amyloid‐like cross‐β‐sheet structures in the presence of Zn^2+^ ions. In particular, the Phe–Zn^2+^ complex demonstrated enzyme‐like catalytic efficiency in pNPA hydrolysis, following Michaelis–Menten kinetics ($k_{\mathrm{cat}}/K_{m}=76.5\;M^{-1}s^{-1}$; Table 4 entry 23). The catalyst retained almost all its activity after five cycles (99%), displaying high thermal stability (up to ∼570 K) at different pH values. Beside this, Phe–Zn^2+^ has also been studied for its catalytic activity in CO_2_ hydration, showing performances that are strongly influenced by pH and temperature (Table 4, entry 24). To broaden the application of Phe‐based assemblies, the same group explored the substitution of Zn^2+^ with Cu^2+^ (yielding Phe‐Cu^2+^ assemblies) to mimic laccase enzymes for environmental remediation [142]. As previously demonstrated for Phe‐Zn^2+^, the formation of the nanosheets occurred along with the coordination of Cu^2+^ ions, creating a 2D layered structure. These Phe‐Cu^2+^ assemblies exhibited significant activity, effectively oxidizing toxic phenolic compounds such as 2,4‐dichlorophenol (2,4‐DP) (Table 4 entry 25). Also, Phe‐Cu^2+^ catalyzed the oxidation of environmental pollutants and catecholamines, with efficiency exceeding that of natural laccase $\left((k_{cat}/K_{m}\right)/MW)=4.0\times 10^{-7}{\left(gl^{-1}\right)}^{-1}s^{-1})$) [142].

**FIGURE 11:**
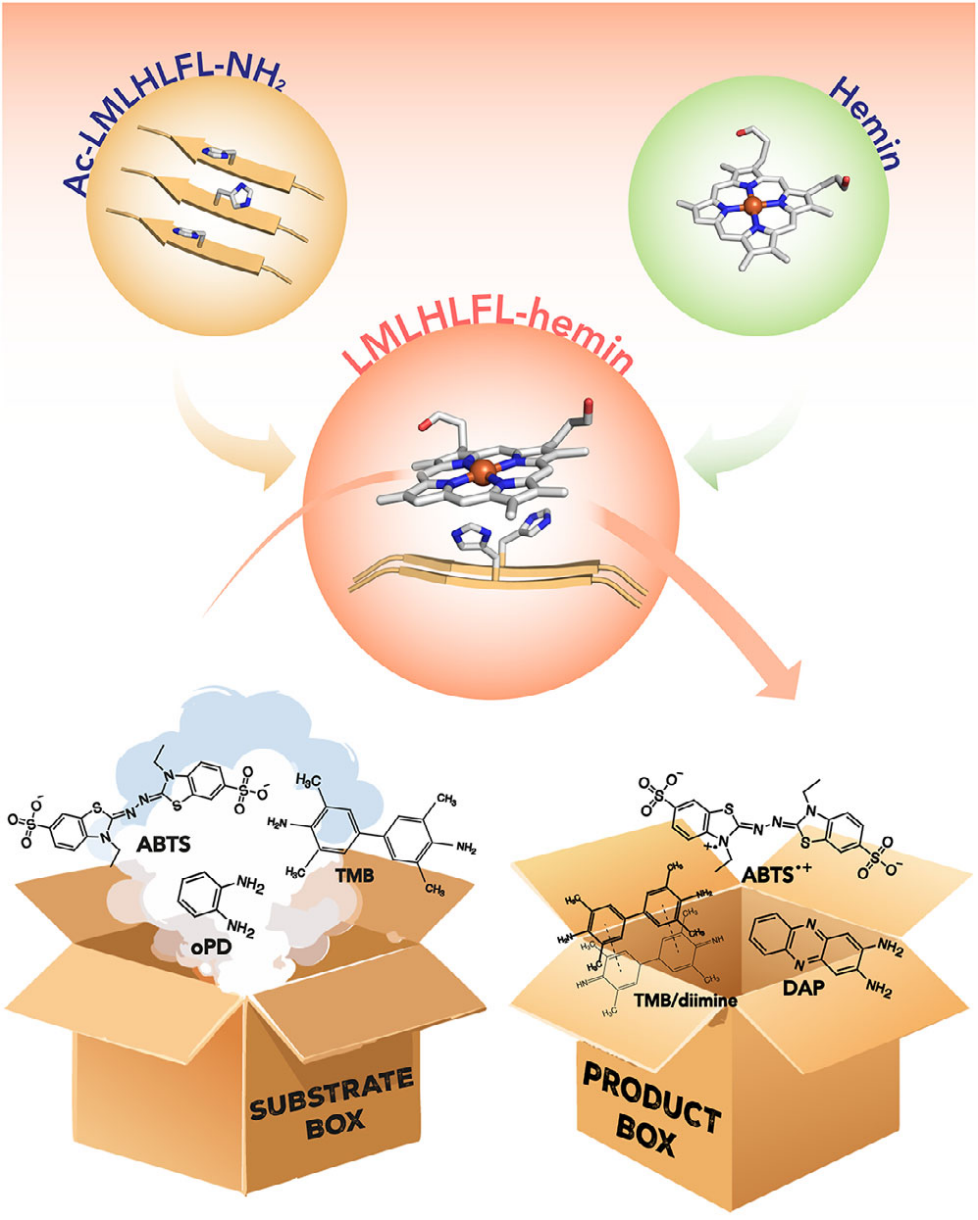
Schematic representation of the catalytic promiscuity of the self‐assembling LMLHLFL‐hemin complex for the oxidation of TMB, ABTS, and oDP in the presence of H_2_O_2_. The structure of the LMLHLFL‐hemin complex is shown at the center, while the various substrates (TMB, ABTS, and oDP) are depicted with their respective molecular structures [145].

Among the variety of metal cofactors studied thus far, heme stands out for its pivotal role in oxidative catalysis. The incorporation of heme into peptide‐based assemblies offers promising ways for the development of catalytic amyloid fibrils in oxidative reactions [143, 144, 145, 146, 147]. In this context, Xu and coworkers [143] incorporated a hemin moiety into a supramolecular hydrogel formed by the self‐assembly of two amino acid derivatives (Fmoc‐Lys and Fmoc‐Phe) with or without the presence of His. The hydrogel formed by all components showed significantly enhanced catalytic activity compared to free hemin, hemin in β‐cyclodextrin, and hemin in polymeric hydrogels. Specifically, it achieved good catalytic efficiency in the oxidation of pyrogallol in the presence of H_2_O_2_, either in aqueous medium, but particularly in toluene (Table 4 entry 26), where k_cat_ value is 136 times higher than free hemin, reaching 60% of the native activity of horseradish peroxidase (HRP). The strategy is generalizable, as similar activities were observed using other Fmoc‐amino acids (Ala, Val, Leu) and different substrates. The amphiphilic and nanoporous structure of the hydrogel facilitates first substrate diffusion between phases, improving catalytic efficiency in organic solvents, and secondly protects hemin from dimerization and oxidative degradation. Notable examples of heme‐binding catalytic amyloids have been provided by Korendovych and coworkers [144, 145, 146], who have explored how amino acid modifications in the *de novo* self‐assembling sequences affect heme binding (Table 4, entries 27 to 31 and Figure 11). In particular [144], the heptapeptide Ac‐LHLHLQL‐NH_2_ was able to bind heme through histidine coordination to iron. To enhance the hydrophobicity and facilitate heme binding, Gln was replaced with Phe, and the His at position 2 with Ile, resulting in the peptide Ac‐LILHLFL‐NH_2_. This assembly not only displayed peroxidase activity but also catalyzed the enantioselective cyclopropanation of 4‐(trifluoromethyl)styrene with ethyl diazoacetate (EDA) with moderate enantiomeric excess (up to 40% ee) (Table 4, entry 27). Subsequently, slight modifications to this peptide enabled to achieve an enhanced peroxidase activity, underscoring the impact of minor structural changes in catalysis. From screening different self‐assembling sequences, LMLHLFL‐hemin emerged as the most active peptide in the oxidation of 3,3’,5,5’‐tetramethylbenzidine (TMB) with H_2_O_2_, achieving a catalytic efficiency of $k_{\mathrm{cat}}/K_{m}^{\mathrm{TMB}}=4.7\cdot 10^{4}\;M^{-1}s^{-1}$ (Table 4 entry 28). Additional substrates were tested, as ABTS (Table 4 entry 29) and o‐phenylenediamine (oDP) (Table 4 entry 30), with the highest efficiency observed for ABTS, yielding $k_{\mathrm{cat}}/K_{m}^{\mathrm{ABTS}}=30\cdot 10^{4}\;M^{-1}s^{-1}$. The hemin‐LMLHLFL assembly was tested for recycling by immobilization onto a PTFE filter membrane, retaining its peroxidase‐like activity over diverse cycles but becoming inactive after 12 substrate passes, likely due to cofactor loss [145]. Finally, Fry group [147, 156] designed innovative nanomaterials starting from peptide amphiphiles able to self‐assemble into nanofibers to bind metalloporphyrins. First, the sequence c16‐AHL_3_K_3_‐CO_2_H was developed, featuring a lysine‐rich hydrophilic head, a leucine‐based β‐sheet‐forming core, and a palmitic acid (c16) tail, with a single histidine serving as the metalloporphyrin binding site [156]. Indeed, zinc protoporphyrin IX was found to effectively bind to the histidine site, as confirmed by UV‐Vis and CD spectroscopies, which indicated strong exciton coupling between chromophores. Subsequently, many analogues of this peptide were developed by mutating residues of the heme binding site (represented by xy) into c16‐xyL_3_K_3_‐CO_2_H [147]. Specifically, y is typically a histidine, the most common heme‐binding amino acid, and x can be either Ala, Met, or His, allowing to obtain heme binding sites with different axial coordination (AA_Heme_, AH_Heme_, HH_Heme_, or MH_Heme_). The resulting peptides were found to effectively bind hemin forming micelles at pH 7 and elongated fibrils at pH 10.5 with a pH‐dependent conformational change. The peroxidase activity of these assemblies was tested in TMB oxidation and, interestingly, was found to be strongly dependent on the nanomaterial morphology. The most striking differences were observed for the AH_Heme_ peptide, displaying enhanced catalytic efficiency in the micellar state compared to the fibrillar state, while the highest catalytic efficiency was displayed by MH_Heme_ micelles (Table 4 entry 32). The changes in reactivity were attributed to the morphological transition from micelles to fibers, which, depending on the peptide sequence, may involve a change in the coordination state of hemin. The above examples highlight how the hierarchical self‐assembly of peptides can generate catalytic microenvironments capable of substrate binding and transformation.

The examples discussed above illustrate how the catalytic properties of de novo amyloids can be tuned through sequence design and cofactor selection, influencing residue arrangement, metal coordination, cooperative assembly, and polymorphism. Both local features (such as the relative positioning of catalytic residues) and supramolecular properties (including assembly morphology) play key roles in determining catalytic efficiency, substrate binding, and reaction selectivity.

Overall, these findings enforce the idea that the catalytic activity of amyloids is influenced by the interplay of multiple factors such as structural surface organization and the precise spatial arrangement of active site residues. As a result, deriving general design principles for highly selective, robust, and efficient catalytic amyloids remains challenging. In this context, high‐resolution structural data, particularly from cryo‐EM, have recently provided critical insights into self‐assembly behavior, enabling more reliable definition and, in principle, improved prediction of supramolecular architectures.

### 4.3 Merging Biocatalysts and Amyloid Fibrils: Novel Catalytic Nanomaterials

Rather than being used for the construction of catalytic sites as described in the previous sections, amyloid scaffolds can be used as platforms for immobilizing natural or artificial enzymes. In this respect, the combination of enzyme engineering, tailored immobilization techniques, and self‐assembled structures is paving the way to the development of a new class of biomaterials with powerful applications. A variety of strategies have been devised to construct catalytic amyloids by conjugating natural enzymes on amyloid fibrils. These approaches foresee the use of fibrils for enzyme immobilization, with the aim to create a suitable environment for the catalyst, preserving their activity while minimizing deactivation, and facilitate their reuse. Further, the colocalization of different enzymes on the amyloid fibrils, by spatially controlled loading, can facilitate cascade transformation thereby increasing the viability of complex biocatalytic processes. The various immobilization strategies exploited so far include enzyme entrapment [157, 158, 159, 160], crosslinking [161, 162], biotin‐streptavidin technology [163, 164, 165, 166], and covalent bonding [167, 168, 169, 170, 171, 172]. Table 5 reports selected examples of new catalytic nanomaterials obtained by merging enzymes or cofactors with amyloid fibrils, grouped on the basis of the immobilization strategy. Details on the catalytic activity and efficiency are also listed. Das and coworkers [157, 158] demonstrated the effectiveness of entrapping different enzymes on the amyloid fibrils to obtain complex cascade reaction networks. (Table 5 entry 1 and 2).

**TABLE 5:** Catalytic nanomaterials based on enzyme and amyloids, grouped respect to the immobilization strategies used.

Entry	Fibrils	Immobilization Strategy	Catalytic Activity	Enzyme/ Cofactor	Substrate	$k_{\mathrm{cat}}/K_{M}$ $\left(M^{-1}s^{-1}\right)$	Ref.
1	Im‐KLVFFAL‐NH_2_	**Entrapping**	Cascade reaction	SOX hemin	SAR GU	/	[157]
2	Ac‐RLVFFAL‐NH_2_ and Im‐RLVFFAL‐NH_2_	**Entrapping**	Alcohol dehydrogenase	ADH	Benzyl alcohol	20	[158]
			Cascade reaction	SOX	N‐methyl glycine	1×10^3^	[158]
				Cat	H_2_O_2_	64×10^4^	
3	Ac‐KLVFFAE‐NH_2_ and Ac‐KLVFFAL‐NH_2_	**Entrapping**	Peroxidase	CytC	Pyrogallol	/	[159]
4	Au@Lysozyme‐GO	**Entrapping**	Peroxidase	HRP	Glucose	/	[160]
5	Insulin	**Cross‐linking**	Oxidase	GOx	Amplex Red Glucose	/	[161]
6	Insulin	**Cross‐linking**	Hydrolase	OPH	Paraoxon	/	[162]
7	Au@Whey protein (WPNFs)	**Biotin‐streptavidin**	Oxidase	GOx	Glucose	/	[163]
8	NYNYNYN QYQYQYQ SYSYSYS	**Biotin‐streptavidin**	Peroxidase	HRP	Luminol H_2_O_2_	/	[164]
			Hydrolase	AP	pNPP H_2_O_2_	/	[164]
			Peroxidase	HRP GOx	Glucose ABTS	/	[164]
9	Sup35 nanowires	**Biotin‐streptavidin**	Peroxidase	HRP	TMB	LoD 0.1 ng/ml	[165]
10	Sup35 nanowires	**Covalent**	Recognition	Protein G	Yersinia pestis F1 antigen	LoD 2 ng/ml	[167]
			Hydrolase	MPH	Methyl parathion	/	[167]
11	SH3 domain	**Covalent**	Oxidase	Cyt	/	/	[168]
12	Ure2 (1–65)	**Covalent**	Carbonic Anhydrase	CA	CO_2_	/	[169]
			Hydrolase	Barnase	Nucleotide	/	[169]
			Transferase	GST	Glutathione	/	[169]
13	Ure2 (1–65)	**Covalent**	Peroxidase	HRP	ABTS	12×10^−9^	[170]
			Phosphatase	AP	pNPP	4×10^−8^	[170]
			Transferase	GST	Glutathione	/	[170]
14	TTR@FeMC6*a	**Covalent**	Peroxidase	FeMC6*a	ABTS	190×10^−3^	[172]

In the cases of biosensors, Limit of Detection (LoD) is also reported, when available.

**FIGURE 12:**
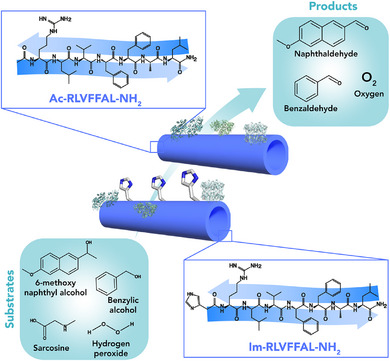
Schematic representation of emergent functionalities generated through a complex divergent cascade activated by enzyme‐loaded amyloid nanotubes.[158] Adapted from Chatterjee A. et al., *Angew. Chem. Int. Ed*. 2022, 61, e202201547. Reproduced with permission of John Wiley and Sons 2022 Wiley‐VCH GmbH.

As a first example, the well characterized Im‐KLVFFAL‐NH_2_ nanotubes (see above **paragraph 4.1**) were used to colocalize sarcosine oxidase (SOX) and the hemin cofactor onto the amyloid surface as a template for a two‐step enzymatic cascade reactions [157]. Notably, the imidazole‐rich nanotube surface is suitable also for a three‐step biocatalytic cascade (Table 5 entry 1). Exploiting the intrinsic hydrolase‐like activity of the imidazole‐exposed amyloid surface, the nanotubes were found able to first catalyze the hydrolysis of methylated sarcosine (Ac‐SAR) to sarcosine (SAR). The SAR was subsequently oxidized by SOX, thus producing hydrogen peroxide (H_2_O_2_) which in turns is used by hemin to catalyze peroxidation of GU, thus allowing a complete three‐step cascade [157]. In a subsequent work, impressive results were obtained on amyloid nanotubes used to host enzymes achieving two functional roles, fluorescent signaling and motility [158]. In particular, nanotubes formed by two types of peptides, one acetylated (Ac‐RLVFFAL‐NH_2_) and one containing imidazole group (Im‐RLVFFAL‐NH_2_) were used (Figure 12 and Table 5 entry 2). The colocalization of enzymes like SOX, catalase (Cat) and alcohol dehydrogenase (ADH) generated fluorescent products while also enabling the nanotubes to move through cascade reactions (Table 5 entry 2). In particular, fluorescence arises from the product of the reaction involving ADH, which binds to the fibrils. On the other hand, cascade reactions involving SOX and Cat, produce O_2_ that trigger fibrils microscopic motility. In this supramolecular system, enzymatic activity in the cascade reactions generates different functionalities, either triggering fluorescence or motility. In a similar approach, the same group [159], prepared CytC‐loaded amyloid nanohybrids based on Ac‐KLVFFAE‐NH_2_ (KE) and Ac‐KLVFFAL‐NH_2_ (KL) sequences. The fibrils were able to non‐covalently bind CytC, unveiling the peroxidase activity of the heme protein [173] in the oxidation of pyrogallol with H_2_O_2_ in organic solvents (Table 5 entry 3). Compared to the activity exhibited by the heme protein in water, the activity of CytC‐loaded fibrils in toluene was dramatically enhanced, showing a 308‐fold and 450‐fold increase for KE and KL fibrils, respectively. The higher catalytic activity was attributed to increased cationic charge density from Lys residues on both surfaces, which enhances the release of the hydrophobic product, purpurogallin. The entrapment approach has also been used to develop innovative nanomaterials based on the combination of amyloid fibrils with very different matrices [160]. The key advantage of this strategy lies in the possibility of obtaining a wide range of protein–amyloid hybrids by leveraging non‐covalent interactions. Along these lines, Li and colleagues [160] explored an innovative entrapment strategy by developing an amyloid‐graphene oxide (GO) platform through the hybridization of 2D GO nanosheets with lysozyme nanofibrils as a carrier for Au nanoparticles and HRP enzyme (Table 5 entry 4). This platform demonstrated high potential as a biosensor, particularly for glucose detection through a colorimetric assay.

Even though the entrapment approaches are easy to perform as they do not require any chemical modifications, either for the catalyst and the fibrils, they may suffer from catalyst leakage and poor stability under reuse conditions. While being a simple procedure for enzyme confinement on nanosupports, cross‐linking methods ensure more stable interactions between amyloid fibrils and enzymes with respect to the entrapment approaches. As reported by the Gerrard group [161, 162], glucose oxidase (GOx) or organophosphate hydrolase (OPH) were successfully attached to insulin amyloid fibrils using glutaraldehyde as a cross‐linking reagent, retaining enzymatic activity. (Table 5 entries 5 and 6). To maximize the potential of these catalytically active functionalized amyloids, GOx@fibrils were further incorporated into polyvinyl alcohol (PVOH) films, demonstrating activity over six months. These films exhibited antibacterial activity against *E. coli*, highlighting the potential of these catalytic nanomaterials for biocatalytic and antibacterial applications [161]. For OPH, immobilization increased enzyme thermostability and generated nanomaterials catalytically active toward the hydrolysis of paraoxon (Table 5, entry 6). The more interesting feature of this system is the reusability, making it ideal for applications such as bioremediation and chemical detoxification [162]. These examples demonstrate the power of using a crosslinking strategy on amyloids for the development of sustainable and biocompatible catalytic platforms. However, enzyme loading and localization by this approach are hard to control, leading to system heterogeneity.

**FIGURE 13:**
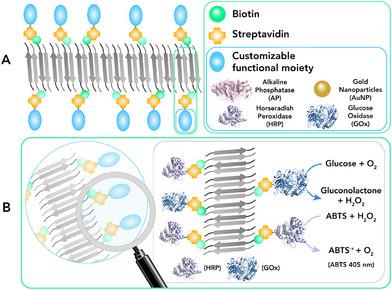
Functionalized prion‐inspired amyloids as scaffold for enzyme immobilization through biotin‐streptavidin technology. (A) Schematic of the adopted approach and (B) tandem reaction of biotinylated peptides in the presence of streptavidin‐HRP and streptavidin‐GOx for glucose detection [164]. Reprinted from Diaz‐Caballero et al., *Biomacromolecules* 2021, 22, 7, 2822–2833. Reproduced with permission of American Chemical Society, Copyright 2021 licensed under CC‐BY 4.0.

Among non‐covalent immobilization strategies, the biotin‐streptavidin system stands out for its exceptionally selective and stable binding affinity, making it highly resistant to harsh chemical and physical conditions and allowing site‐specific conjugation. Thus, this non‐covalent interaction has been widely applied to engineer amyloid fibrils for immobilization of enzymes, proteins, antibody, and small molecules, useful in various application fields, including biosensing [163, 164], immunodetection [165], and nanotechnology [166]. The advantage of this strategy is the possibility of engineering biotinylated amyloid‐like nanofibers for easy subsequent functionalization with streptavidin‐conjugated entities (like enzymes or nanoparticles), thus providing multivalent nanomaterials as biosensing platforms and catalytic assemblies (Figure 13A). In this context, Gerrard and coworkers [163] reported an adaptable method for the multi‐functionalization of whey protein nanofibrils (WPNFs), enabling their use as biosensors (Table **5** entry 7).

**FIGURE 14:**
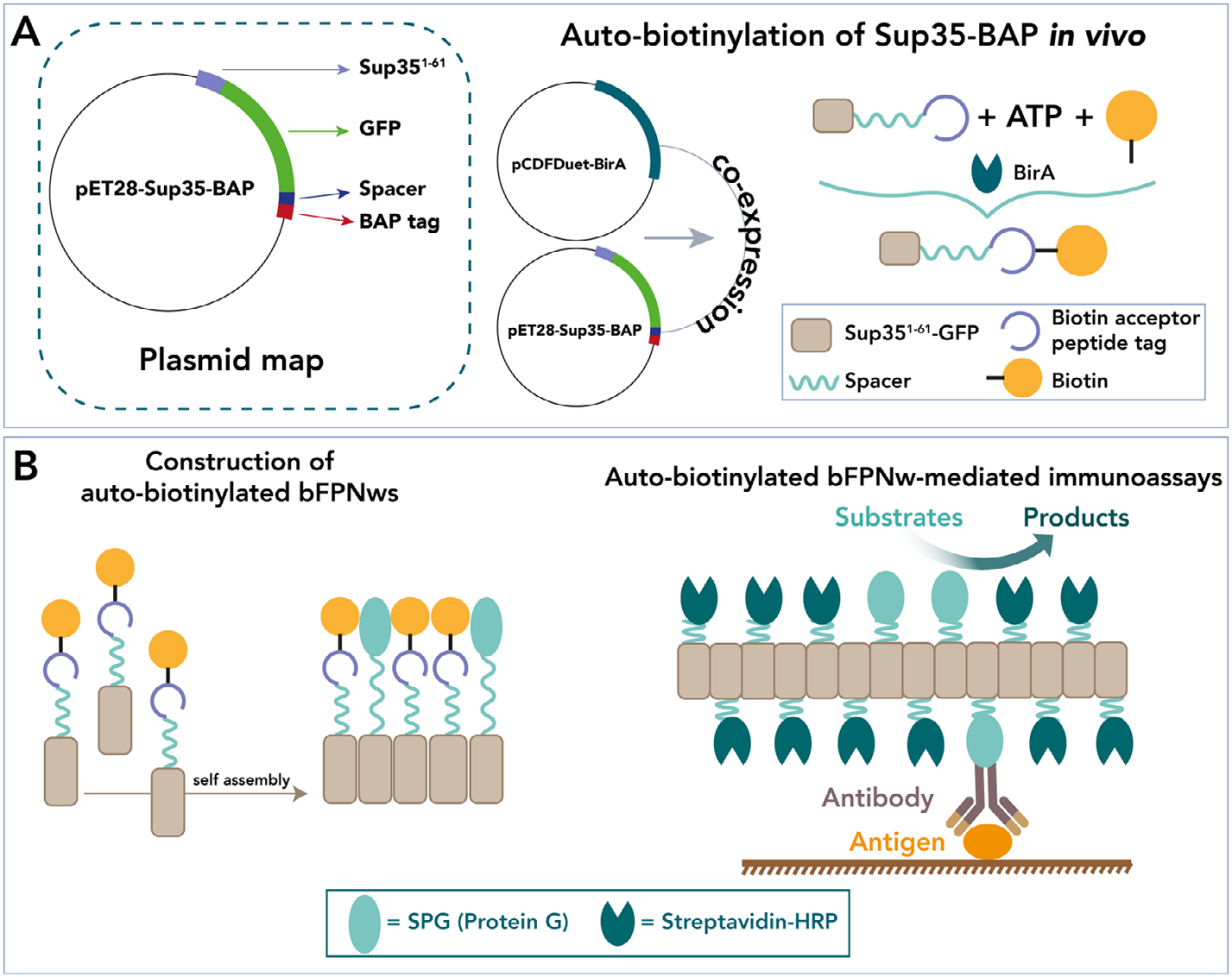
(A) Schematic illustration of the self‐assembled, auto‐biotinylated fusion protein Sup35‐BAP. The pET28‐Sup35‐BAP plasmid map and in vivo auto‐biotinylation of Sup35‐BAP are shown. (B) Construction of auto‐biotinylated bifunctional protein nanowires (bFPNws) and their application in bFPNws‐mediated immunoassays. An indirect ELISA was performed to assess the antibody‐capturing and streptavidin‐binding capabilities of the auto‐biotinylated bFPNws using the F1 antigen as a model analyte [165]. Adapted from Men D. et al., *Biosens. Bioelectron*. 2010, 26(4), 1137–1141. Reproduced with permission from Elsevier. Copyright 2010, Elsevier.

The conjugation was achieved through biotin groups on the WPNF surface and streptavidin‐modified components, as quantum dots and GOx. These functional WPNFs were used for the development of a glucose biosensor platform by immobilizing the GOx‐nanofibrils onto gold electrodes. The same conjugation strategy was used by Ventura and coworkers [164], who engineered prion‐like heptapeptides with biotin moiety, creating a scaffold for streptavidin‐conjugated gold nanoparticle (AuNPs), HRP, GOx, and alkaline phosphatase (AP) (Table 5 entry 8 and Figure 13A). In particular, the biotinylated fibrils (Biotin‐NY7, Biotin‐QY7, and Biotin‐SY7) self‐assemble rapidly and react with streptavidin‐liked enzymes (*i.e*., HRP and GOx) to develop a biosensor for the glucose detection (Figure 13B). Zhang and coworkers [165] developed bifunctional protein nanowires (bFPNws) based on the self‐assembly prion domain of Sup35, to which protein G and a biotin acceptor peptide (BAP) were genetically fused. These nanowires were auto‐biotinylated in vivo through the coexpression of the biotin ligase BirA, avoiding the need for other chemical modification. The resulting biotinylated nanowires were able to bind streptavidin‐HRP conjugates, thus allowing the sensitive detection of the *Yersinia pestis* F1 antigen (Table 5 entry 9 and Figure 14). Remarkably, this bioengineered system achieved an improved detection limit (0.1 ng/mL) compared to conventional ELISA methods.

**FIGURE 15:**
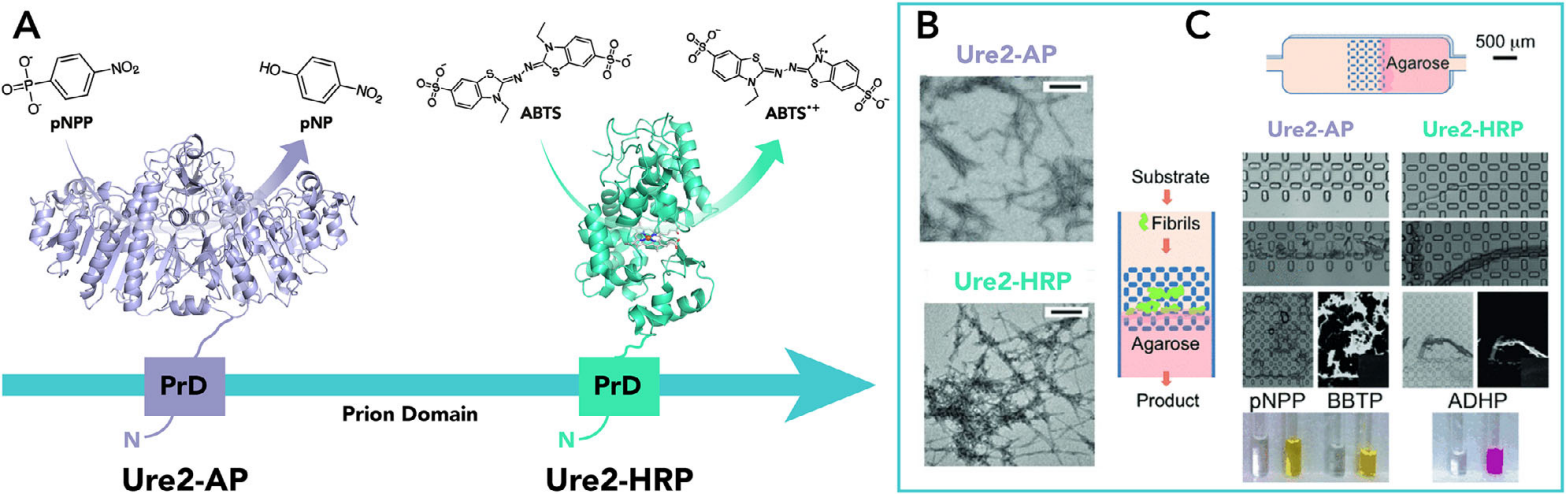
Schematic overview of (A) the fusion approach between a prion domain and AP/HRP enzymes, (B) TEM images of chimeric Ure2 fibrillar aggregates (scale bars 200 nm), and (C) the use of chimeric fibrils in a continuous‐flow microreactor [170]. Adapted from Perret S. et al., *ChemCatChem*, 2014, 6, 1961–1968. Reproduced with permission of Wiley‐VCH Verlag GmbH and Co. KGaA. 2014 The Authors.

Covalent binding of enzymes and proteins to amyloid fibrils has been fruitful for the development of catalytic nanomaterials. By exploiting protein fusion techniques [167, 168, 169, 170, 171], Zhang group [167] reported the design and expression of bifunctional protein nanowires, built by seeding‐induced self‐assembly of Sup35 prion domain to produce a sensitive immunoassay (Table 5 entry 10). Methyl‐parathion hydrolase (MPH) and protein G were genetically fused with this protein. The resulting nanowires presented a high enzyme‐to‐protein G ratio, leading a significant increase in enzymatic signal, thus enhancing the sensitivity for the Yersinia pestis F1 antigen detection. Moreover, Barker, Dobson, and coworkers [168] developed an amyloid chimera by fusion of a b‐type cytochrome with an amyloid‐forming domain derived from a tandem repeat of SH3 proteins (Table 5 entry 11). The resulting amyloid fibrils were shown to effectively bind metalloporphyrins, with TEM micrographs revealing a density of 2.6 CytC units per nanometer. The high concentration of enzyme on the fibril surface suggests potential application for rapid electron transfer in assembled molecular wires. Following a similar approach, Baxa group [169] explored the effects of fusing the Ure2 prion domain to enzymes, such as CA, barnase, glutathione S‐transferases (GST), and proteins like GFP. Their findings indicate that GST and GFP fusions maintained significant activity (∼80% and ∼130%, respectively, Table 5 entry 12), while barnase and CA fusions showed minimal activity. This suggests that fibrils inactivation likely occurs due to steric hindrance rather than conformational changes affecting the functional domains. The same Ure2 prion domain was used by Sawyer, Perrett and coworkers [170] to construct a protein chimera containing enzymes like alkaline phosphatase (AP) and HRP (Table 5 entry 13 and Figure 15).

The catalytic evaluation of enzyme‐decorated fibrils, using pNPP for alkaline phosphatase (AP) and ABTS for HRP, revealed distinct effects from amyloid fusion. For AP, amyloid fusion decreased the substrate affinity and the turnover rate (k_cat_) by approximately 10‐fold in comparison to its wild type. Instead, a different behavior was observed for HRP, which demonstrates a notable decrease in activity ($k_{\mathrm{cat}}/K_{M}^{\mathrm{ABTS}}=120\;nM^{-1}s^{-1}$ vs $k_{\mathrm{cat}}/K_{M}^{\mathrm{ABTS}}=1440\;nM^{-1}s^{-1}$ for WT HRP), likely due to a steric hindrance, which hampers the substrate access to the active site. Despite the reduction of the catalytic activity of the immobilized enzyme compared to the freely diffusing, the prion domain of Ure2 appeared as a good scaffold for the preparation of catalytically active nanomaterial that can be recycled and reused either in batch or continuous flow reactors (Figure 15C).

**FIGURE 16:**
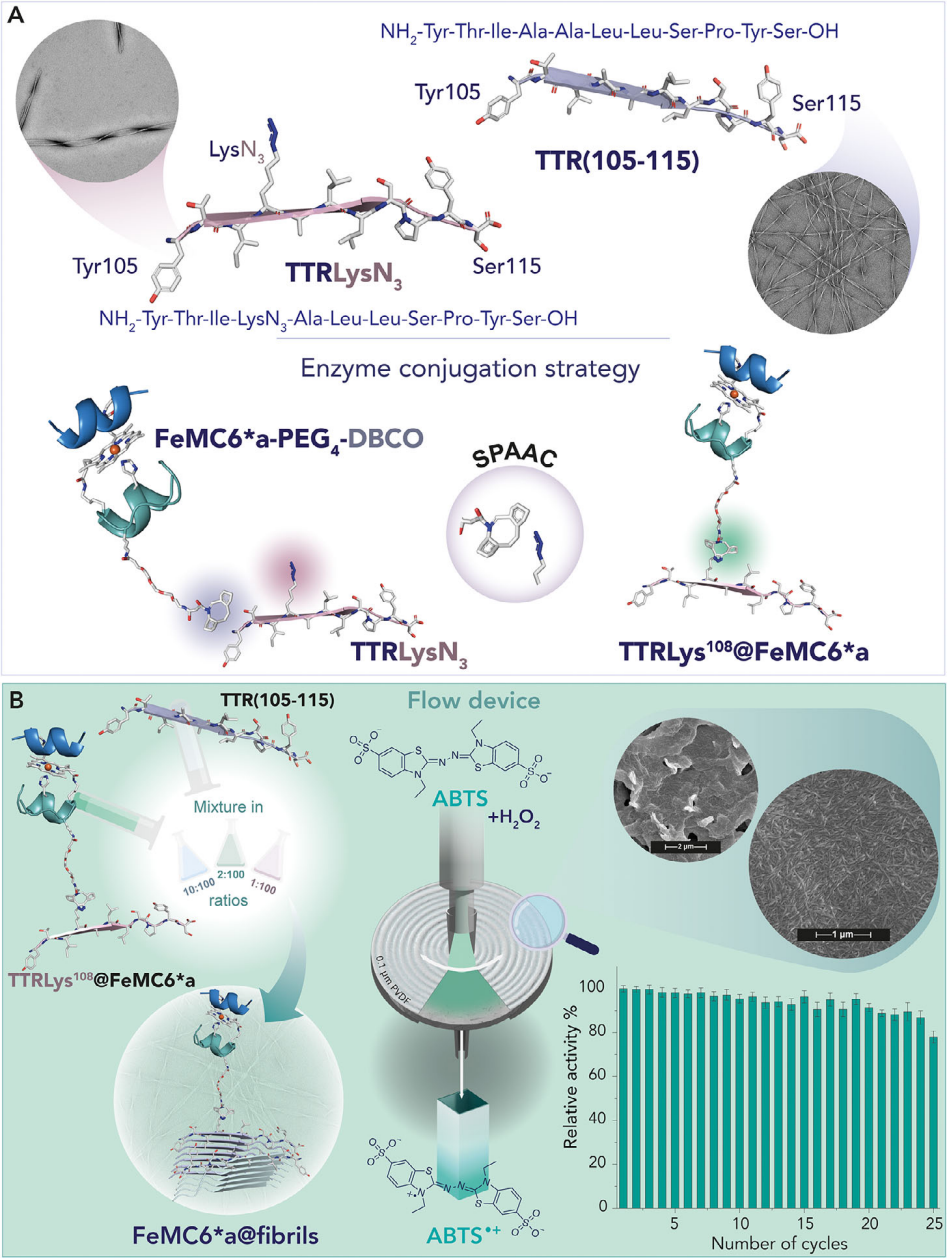
Schematic representation and characterization of the catalytic nanomaterial system obtained by conjugating an artificial heme‐peroxidase to amyloid fibrils. (A) Selected sequences of the TTR(105–115) monomer, the modified analogue TTRLysN_3_, and a schematic illustration of the functionalization of TTRLysN_3_ with FeMC6*a, yielding TTRLys^108^@FeMC6*a conjugate. (B) Synthesis of FeMC6*a@fibrils and schematic representation of their self‐assembly with TTR(105–115) peptide at different ratios. Membrane‐based reactor for ABTS oxidation, SEM images showing FeMC6*a@fibrils deposited on a PVDF membrane, and histogram of absorbance at 660 nm corresponding to oxidized ABTS produced in each reuse cycle [172]. Adapted from Esposito, A. et al., *ACS Appl. Mater. Interfaces* 2024, *16* (34), 45371–45382. Reproduced with permission of American Chemical Society, Copyright 2024.

Merging the unique features of amyloid fibrils with artificial biocatalysts can further empower the field. In this context, our group [172] developed a catalytic amyloid nanomaterials through the conjugation, by click chemistry, of a miniaturized artificial peroxidase, namely FeMC6*a [174, 175, 176], to a self‐assembling amyloidogenic peptide derived from human transthyretin, TTR(105−115). This peptide sequence was previously used by Knowles and coworkers [177] for anchoring by *click chemistry* approach a fluorescent protein. Differently from this study, in our approach the peptide sequence of TTR(105−115) was modified by substituting Ala at position 108 with *ε*‐azido‐Lys (LysN_3_) (affording TTRLysN_3_). This substitution did not affect the peptide's ability to assemble into fibrils and introduced a suitable functionality for conjugating the artificial FeMC6*a derivatized with a pegylated aza‐dibenzocyclooctyne (FeMC6*a‐PEG_4_@DBCO) (Table 5 entry 14 and Figure 16), thus affording TTRLys^108^@FeMC6*a. Mixing this with unmodified TTR(105−115) allowed the generation of enzyme‐loaded amyloid fibrils, namely FeMC6*a@fibrils, which possess unique characteristics merging the morphology of the unfunctionalized nanomaterial with the catalytic activity of the artificial enzyme. Catalytic studies using ABTS as a model substrate and H_2_O_2_ as an oxidizing agent reveal that the FeMC6*a@fibrils retain the catalytic performances of the freely diffusing enzyme. Interestingly, the activity was found to be dependent on the ratio of FeMC6*a‐modified to unmodified peptide, with the highest initial reaction rate for the 2:100 ratio, respect to all the screened nanoconjugates. To expand the applicability of this developed nanomaterial, FeMC6*a@fibrils were integrated into a flow catalytic system using a membrane filter. Within this flow‐reactor setup, the catalytic nanomaterial performed multiple reaction cycles, demonstrating stability and reusability over time. The added value of this nanomaterial is related to the small size of the catalyst with respect to its natural counterpart (HRP), that allow to reduce the drawback of catalyst overcrowding onto the fibril surface. Further, the established catalytic promiscuity of FeMC6*a and its analogues may offer the possibility of expanding toward different substrates and applications. The selected examples described above showcase that the combination of natural or artificial enzymes with amyloid nanostructures provides a powerful toolbox for the design of efficient, durable, and multifunctional catalytic systems for a variety of applications ranging from enzyme recycling, flow chemistry, biosensing to fibrils motility. Although several nanostructures, including metal nanoparticles and mesoporous silica, have been explored as immobilization supports with enhanced surface‐area‐to‐volume ratio, they still present notable limitations. These include poor dispersion in solution, a diffusion barrier for substrates, limited immobilization efficiency, and difficulties with reuse. In contrast, amyloid fibrils offer significant advantages over conventional nanomaterials. Their elongated fibrillar architecture provides a highly accessible surface area, enabling high enzyme loading compared with flat or spherical supports. Unlike many metal‐, carbon‐, or polymer‐based materials, amyloid fibrils can be directly functionalized through their amino acid side chains, allowing robust covalent enzyme attachment without the need for costly and time‐consuming multi‐step functionalization procedures. Moreover, they are easily dispersed in aqueous solutions and can be entrapped within porous matrices, facilitating continuous operation in flow‐based catalytic systems.

### 4.4 Catalysis Driven by Amyloid Hydrogel Architecture

**FIGURE 17:**
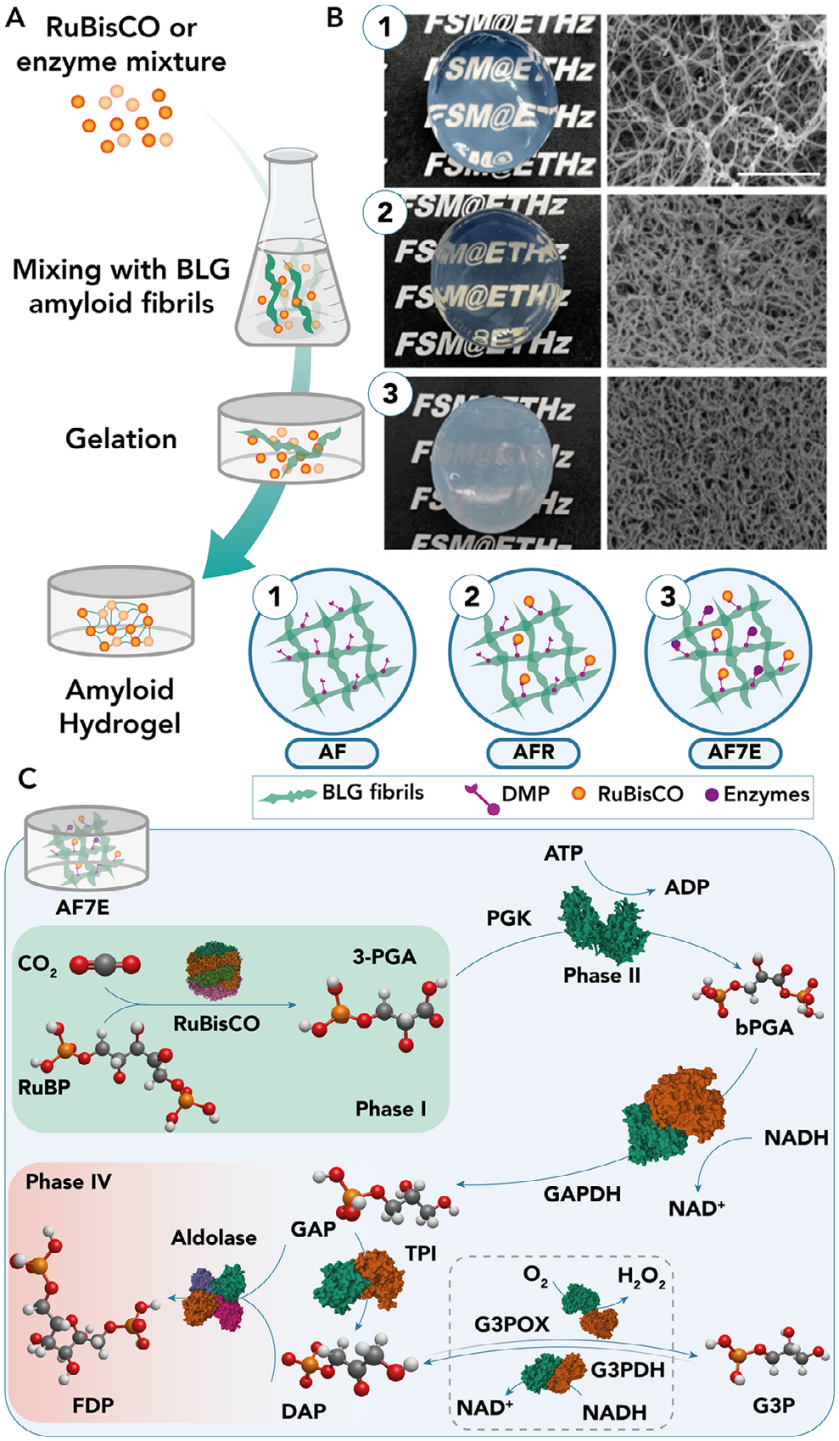
Formation of a multienzyme hydrogel. A) Schematic of enzyme immobilization in amyloid fibril hydrogels. B) SEM micrographs of the dried samples (scale bar: 1 µm) and amyloid‐enzyme interactions. In detail, AF (1) is a pristine amyloid hydrogel with dimethyl pimelimidate dihydro‐chloride (DMP) cross‐linkers on the fibrillar network, AFR (2) is the amyloid hydrogel immobilized with RuBisCO and AF7E (3) is the hydrogel immobilized with the enzyme mixture. C) Enzymatic reactions in AF7E with immobilized enzymes [181]. Adapted from T. Jin, et al., ACS Nano 2025, 19 (4), 4820–4829. Reproduced with permission of American Chemical Society.

Many peptides and proteins can assemble into fibrillar networks that form hydrogels by trapping large amounts of solvent [178, 179]. This process is driven mainly by noncovalent interactions or, in some cases, by intentionally introduced covalent crosslinks. This tendency is peculiar to natural amyloid sequences, ranging from pathological to biocompatible proteins [180]. Within the field of catalytic amyloids, this hierarchical organization provides an added value for their applicability. Indeed, hydrogels provide a confined microenvironment that brings immobilized enzymes into close spatial proximity. This proximity effect facilitates efficient substrate channeling between successive enzymatic steps, reducing diffusion distances and minimizing the loss of intermediates. As a result, hydrogels can effectively enable cascade or multistep reaction pathways within a confined matrix, enhancing reaction efficiency and mimicking the organization found in natural biological systems. The most representative example of this approach was recently reported by Mezzenga and colleagues [181], who effectively achieved coimmobilization of seven enzymes within β‐lactoglobulin‐based hydrogels through a cross‐linking strategy (Figure 17). In detail, ribulose 1,5‐biphosphate carboxylase/oxygenase (RuBisCO), 3‐phosphoglyceric phosphokinase (PGK), α‐glycerophosphate dehydrogenase (GAPDH), triosephosphate isomerase (TPI), glyceraldehyde 3‐phosphate dehydrogenase (G3PDH), glycerol 3‐phosphate oxidase (G3POX), and catalase were crosslinked in a one‐step process to a hydrogel of β‐lactoglobulin fibrils. The obtained catalytic nanomaterial (named AF7E) enabled the conversion of CO_2_ into fructose through an enzymatic cascade, mimicking natural photosynthesis. Compared to free enzymes, the hydrogel shows higher 3‐phosphoglycerate production while maintaining ∼90% enzyme activity after 60 days. Further, AF7E hydrogel was totally biodegradable in the presence of pepsin, leading to the loss of the β‐sheet structure. The same group developed a hybrid nanomaterial using lysozyme amyloid fibrils (Lys‐AFs) as a template for the synthesis of nanozymes (Lys‐AFs‐Ceria‐GOx) [182], combined with GOx for diabetic wound healing. Incorporated into hydrogel microneedles, this system exhibited superoxide dismutase (SOD)‐like and catalase (CAT)‐like activities, reducing oxidative stress, promoting angiogenesis, and ultimately accelerating wound healing. The hydrogel provided a controlled release of the nanozymes to the wound while acting as a supportive matrix and preventing their inactivation. Additionally, the amyloid hydrogel displayed antimicrobial properties while supporting cell adhesion and tissue regeneration. Lastly, Wang and coworkers [183] developed a multifunctional nanomaterial by fusing the Sup35 prion domain with the SpyCatcher protein, resulting in the formation of fibrils able to covalently bind any protein or enzyme functionalized with SpyTag peptide. By further incorporating a mussel foot protein‐mimetic peptide (Mfp3Sp) at the N‐terminus, they engineered an amyloid‐based hydrogel with enhanced adhesion and mechanical stability. This strategy enabled the colocalization of glucose dehydrogenase (GDH) and Cytochrome P450 (P450) within the hydrogel, affording a dual enzymatic system within the nanomaterial capable of promoting indole conversion to indigo coupled to NADPH recycling. The dense fibrillar network of the hydrogel promotes substrate channeling and increases local substrate concentration, enhancing enzymatic efficiency. When the hydrogel was coated onto SiO_2_ microparticles, fibrils showed up to sixfold higher activity (in terms of initial reaction rate) with respect to the free enzymes and retained 80% of their activity after five cycles. Collectively, these examples highlight how the hydrogel state offers a versatile platform for organizing enzymes in close spatial proximity, a key factor enabling efficient substrate channeling and the integration of multistep reaction pathways. At the same time, the confined environment may impose limitations related to mass transport, fibril packing density, or long‐term mechanical stability. Such constraints can be mitigated through controlled fibril architecture or the development of hybrid systems, allowing the proximity effect to be retained while preserving accessibility and structural robustness.

## 5 Summary and Outlook

Over the past fifty years, amyloids have evolved from being recognized as pathological aggregates to serve as a source of innovation for catalysis and nanotechnology [184]. In particular, principles that govern amyloid self‐assembly have been successfully exploited to design peptide‐based nanostructures able to achieve specific functions [1]. In this review, we have illustrated the potential of amyloids, tracing the path from their biological roles to their catalytic activity. Natural amyloid sequences were found not solely to serve as structural scaffolds but also to promote a variety of chemical reactions, performing esterase, phosphatase, and oxidative activities [97, 98, 99, 100, 101, 102]. These systems were repurposed into bioinspired amyloids, where natural sequences were rationally modified to mimic the active sites of natural enzymes. In this respect, numerous studies focused on isolating short fragments from Aβ42, capable of self‐assembling while displaying enzyme‐like activities [121]. Rather than depending on natural sequences, *de novo* design has been applied for the construction of completely new sequences from scratch, enabling to precisely control the structural and catalytic properties of the resulting nanomaterials. In this context, a key approach has involved the incorporation of critical residues, such as His, Ser, and Asp, into minimal peptide sequences, achieving catalytic activity in the presence or absence of cofactors [20, 34]. In parallel, we have also provided an overview of the immobilization strategies to anchor enzymes onto amyloid fibrils. These have enabled the construction of nanomaterials that combine the selectivity and efficiency of enzymatic catalysis with the robustness and recyclability of heterogeneous systems [110]. In this context, the ability of amyloid fibrils to form hydrogel networks has been exploited to enhance and regulate catalytic activity, enabling complex multistep reactions through multiple enzyme immobilization.

Despite the impressive progress achieved so far, several challenges still limit the application of catalytic amyloids and the understanding of their reaction mechanisms. Indeed, the repertoire of transformations promoted by these systems remains relatively narrow, and their catalytic efficiencies, although significant, are often far from those of their natural counterparts. In most cases catalytic amyloids cannot compare inorganic catalysts in terms of reaction variety, especially for C‐C bond formation, redox reactions, or multi‐electron processes. Indeed, the current reaction repertoire remains largely confined to ester hydrolysis and oxidation, with very limited examples of synthetically relevant chemistry. The bottleneck is the field strong reliance on model chromogenic substrates (*i.e*., pNPA and ABTS). Although useful for detecting catalytic activity, these substrates poorly represent realistic biochemical or industrial transformations. However, catalytic amyloids may outperform natural enzymes or inorganic catalysts in settings that demand high robustness, low cost, and resistance to harsh conditions. Their remarkable stability toward heat, pH, and solvents allows catalytic reactions under conditions that deactivate most enzymes. Moreover, the simplicity of self‐assembly from short peptides and their tunable surface chemistry can offer advantages when biocompatibility or modularity is required. Beyond this, future progress will depend on addressing some practical aspects. For example, the scale‐up of fibril production remains challenging due to batch‐to‐batch variability and limited control over fibril polymorphism, which could complicate the reproducibility of their macroscopic properties and catalytic activities. Although fibrils exhibit remarkable chemical stability and mechanical robustness in vitro, their long‐term storage stability and behavior under physiological conditions remain not yet investigated. Moreover, for amyloids derived from natural sequences, attention must be paid to possible toxicity or immunogenicity, underscoring the importance of assessing their behavior in cellular or in vivo environments before any broader application. The combination of mechanistic insight and practical considerations can propel the field beyond model reactions and reveal their full potential in more complex and relevant transformations. [185] In particular, machine‐learning approaches hold great promise in accelerating sequence discovery and optimization [186]. Future developments could lead to identify new combinations of residues and motifs displaying entirely new reactivities and/or enhanced catalytic performance. In this context, the coassembly of different peptide building blocks bearing distinct reactive groups could drive amyloid systems toward more enzyme‐like behavior by generating more complex and functionally rich microenvironments for substrate recognition and transformation. Furthermore, engineering multi‐functional amyloid fibrils could increase the complexity of cascade reactions, broadening their applicability in biosensing, environmental, or medical fields. For instance, the incorporation of photoactive components along with catalytic units into amyloid scaffolds may enable new types of light‐driven processes [187]. In summary, combining sequence selection with diverse functional moieties could advance the development of stable, efficient nanomaterials to address challenges in green and sustainable chemistry.

## Conflicts of Interest

The authors declare no conflict of interest.

## Data Availability

Data sharing is not applicable to this article as no new data were created or analyzed in this study.
